# Precision Surface Microtopography Regulates Cell Fate via Changes to Actomyosin Contractility and Nuclear Architecture

**DOI:** 10.1002/advs.202003186

**Published:** 2021-01-29

**Authors:** James Carthew, Hazem H. Abdelmaksoud, Margeaux Hodgson‐Garms, Stella Aslanoglou, Sara Ghavamian, Roey Elnathan, Joachim P. Spatz, Juergen Brugger, Helmut Thissen, Nicolas H. Voelcker, Victor J. Cadarso, Jessica E. Frith

**Affiliations:** ^1^ Department of Materials Science and Engineering Monash University Wellington Road Clayton Victoria 3800 Australia; ^2^ Centre to Impact Antimicrobial Resistance – Sustainable Solutions Monash University Clayton Victoria 3800 Australia; ^3^ Department of Mechanical and Aerospace Engineering Monash University Wellington Road Clayton Victoria 3800 Australia; ^4^ Melbourne Centre for Nanofabrication Victorian Node of the Australian National Fabrication Facility Clayton Victoria 3168 Australia; ^5^ Monash Institute of Pharmaceutical Sciences Monash University 381 Royal Parade Parkville Victoria 3052 Australia; ^6^ Commonwealth Scientific and Industrial Research Organisation (CSIRO) Clayton Victoria 3168 Australia; ^7^ Department of Cellular Biophysics Max Planck Institute for Medical Research Jahnstraße Heidelberg D‐69120 Germany; ^8^ Heidelberg University Institute for Molecular Systems Engineering (IMSE) Heidelberg D‐69120 Germany; ^9^ Max Planck School Matter to Life Germany; ^10^ Microsystems Laboratory École Polytechnique Fédérale de Lausanne (EPFL) Lausanne 1015 Switzerland

**Keywords:** mechanotransduction, mesenchymal stem/stromal cells, microtopography, osteogenesis

## Abstract

Cells are able to perceive complex mechanical cues from their microenvironment, which in turn influences their development. Although the understanding of these intricate mechanotransductive signals is evolving, the precise roles of substrate microtopography in directing cell fate is still poorly understood. Here, UV nanoimprint lithography is used to generate micropillar arrays ranging from 1 to 10 µm in height, width, and spacing to investigate the impact of microtopography on mechanotransduction. Using mesenchymal stem cells (MSCs) as a model, stark pattern‐specific changes in nuclear architecture, lamin A/C accumulation, chromatin positioning, and DNA methyltransferase expression, are demonstrated. MSC osteogenesis is also enhanced specifically on micropillars with 5 µm width/spacing and 5 µm height. Intriguingly, the highest degree of osteogenesis correlates with patterns that stimulated maximal nuclear deformation which is shown to be dependent on myosin‐II‐generated tension. The outcomes determine new insights into nuclear mechanotransduction by demonstrating that force transmission across the nuclear envelope can be modulated by substrate topography, and that this can alter chromatin organisation and impact upon cell fate. These findings have potential to inform the development of microstructured cell culture substrates that can direct cell mechanotransduction and fate for therapeutic applications in both research and clinical sectors.

## Graphical abstract

Specific micropillar array geometries enhance mesenchymal stem cell (MSC) osteogenesis by modulating nuclear architecture. By engineering precise surface topographies that interact with the cells, nuclear and chromatin architecture can be changed in a manner dependent upon actomyosin contractility that promotes MSC osteogenesis. This provides new insights into how force transmission across the nuclear envelope can be modulated by substrate topography.

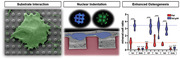

## 1 Introduction

Understanding how mechanical or physical cues in the extracellular environment invoke a cellular response has developed as a key topic of interest in recent years. It is well documented that cells generate traction forces induced by cytoskeletal tension,^[^ ^1^, ^2^ ^]^ and that through the application of microtopographic growth substrates, cellular processes such as attachment and fate determination can be modulated.^[^ ^3^ ^]^ Mesenchymal stem/stromal cells (MSCs) are a preferred model to study extracellular environment effects due to their high mechanosensitivity. For MSCs, traction forces enable the translation of physical cues from focal adhesion sites to the nuclear compartment, facilitating both gene and protein level modulation that enables the adaptive cell response.^[^ ^4^, ^5^, ^6^ ^]^ There has been significant progress in understanding the mechanosensitive mechanisms which underpin MSC self‐renewal and differentiation, due to their importance in immunomodulation, paracrine signaling, tissue engineering, and regenerative medicine. It has been demonstrated that maintaining appropriate MSC mechanosignaling (and thus phenotype) is critical to effective MSC expansion, development, proliferation, growth, survival,^[^ ^7^, ^8^ ^]^ tissue integration, and paracrine signaling.^[^ ^9^, ^10^, ^11^, ^12^ ^]^ Perturbations to this mechanosignaling result in abnormalities to cellular function and tissue homeostasis.^[^ ^13^, ^14^ ^]^ Current tissue engineering strategies focus on replicating in vivo‐like environments, to reproduce the native mechanotransducive signaling events arising from exposure to soluble growth factors, shear stress, hydrostatic pressures, compression, tension, and the mechanical stiffness of the microenvironment.^[^ ^5^, ^15^, ^16^, ^17^, ^18^, ^19^ ^]^ However, modulation of substrate topography has also been demonstrated to regulate both cell and nuclear geometry, alongside gene and protein expression profiles.^[^ ^9^, ^20^, ^21^ ^]^ Most recently, Zhang et al. demonstrated that by simply altering the microtopography of hMSC growth substrates, the expression profiles of the International Society for Cellular Therapy (ISCT)‐defined MSC marker CD90 was significantly downregulated,^[^ ^22^ ^]^ demonstrating the intricate interplay between substrate topography and cell fate.

The design and fabrication of programmable surface micro/nanotopographies to modulate cell behavior was pioneered by Curtis et al.,^[^ ^23^, ^24^ ^]^ and has since been performed using a variety of techniques, often presenting a trade‐off between speed/scale of surface patterning and resolution of the final features. For example, the use of solvent–non‐solvent fabrication methods and electrospinning has been widely used in cell biology research to understand the roles of topography on cell function,^[^ ^25^, ^26^ ^]^ but are limited by the inability to produce pre‐defined high‐resolution features. Micro electro mechanical systems (MEMS) technologies have emerged as alternative fabrication processes for the development of bio‐inspired micro‐topographies, enabling the fabrication of precisely designed, high resolution structures to more accurately understand how cells perceive and respond to topographical features.^[^ ^4^, ^27^, ^28^ ^]^

Despite a large number of studies demonstrating the power of substrate micro and nanotopographic cues for orchestrating cell function, the underlying mechanisms that drive cellular responses are still poorly understood. Functional changes in cell behavior are ultimately driven by alterations in gene transcription, requiring mechanical information to be transmitted to the nucleus through a combination of transcription factor shuttling (e.g. via YAP) and direct force transmission across the nuclear envelope (NE). Systematic studies consistently identify dynamic morphological changes to the nuclear compartment regulated by micro/nanopatterned topographical cues.^[^ ^29^, ^30^, ^31^ ^]^ As nuclear architecture is hierarchical, with chromosome territories occupying largely distinct sites in the nucleus determined by size or gene richness,^[^ ^32^ ^]^ these changes in nuclear phenotypes may hold the key to understanding how and why cell fate can change in response to topographic features. Peripheral DNA, situated around the nuclear exterior, is composed of transcriptionally inactive heterochromatin, while the more frequently expressed euchromatic sequences are primarily centrally localized.^[^ ^33^, ^34^, ^35^ ^]^ The distinct segregation of heterochromatin to the nuclear exterior has been suggested to safeguard nuclear architecture in response to dynamic mechanical stresses.^[^ ^22^, ^36^, ^37^ ^]^ Heterochromatin has been further demonstrated to bear the force from cytoskeletal components via direct mechanical coupling between the cytoskeleton, embedded nuclear envelope proteins, and the nuclear lamina.^[^ ^38^ ^]^

We suggest that in‐depth characterization of how microtopographies modulate nuclear architecture may be critical to defining the mechanotransductory pathways that regulate both phenotypic and genotypic changes to cultured cells.

Recent research has hinted at a correlation between enhanced osteogenesis of MSCs and substrate topography.^[^ ^3^, ^39^ ^]^ Thus, enhancing our understanding of the precise cellular mechanisms which regulate this process will refine the way we approach biomaterial development, in which cell adhesion, fate determination, and implant osseointegration may be determined by modulation of topographic features.

Here we present a micropatterned library consisting of square pillar designs and demonstrate their capacity to reshape nuclear architecture via direct tension across the NE. We determine precise microstructural features able to influence MSC fate determination and further validate how these topographical changes regulate chromatin re‐organization, gene activity, and subsequent osteogenic fate. Our results point to a previously undefined mechanism by which mechanotransductory signaling can be effectively harnessed using microtopographies for future clinical settings such as translation to bone implant technologies; and how this paradigm might reduce the reliance on biological factors such as growth factors.

## 2 Results and Discussion

### 2.1 Fabrication of Biocompatible High‐Resolution Microstructures

**Figure 1:**
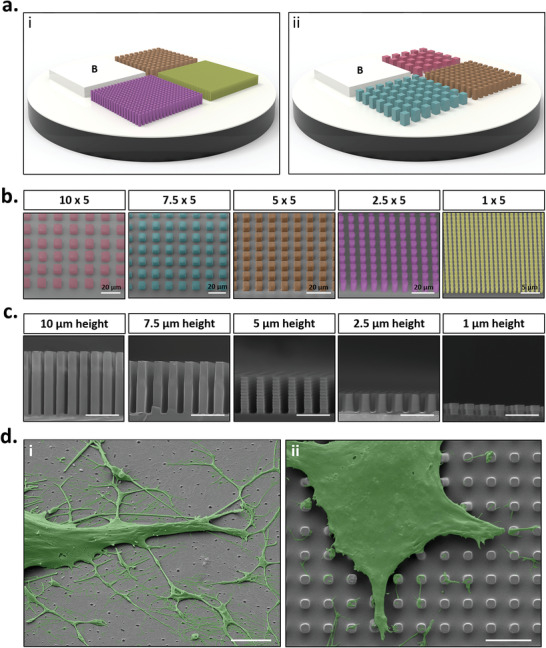
Fabrication of high resolution micropatterned substrates. a) Schematic representation of microfabricated grid platforms containing micropillar designs; i) Blank (flat controls), 1 × 5 (yellow), 2.5 × 5 (purple) and 5 × 5 (orange), or ii) Blank (flat control), 5 × 5 (orange), 7.5 × 5 (blue), and 10 × 5 (red). b) Scanning electron micrographs of micropatterned designs. Grid designs are described in the format of micropillar width and spacing × micropillar height. c) Scanning electron micrographs of micropillars in cross‐section. Heights range from 1 to 10 µm. d) Representative false coloured scanning electron micrographs highlighting phenotypic differences between MSCs cultured on i) flat (control) substrates and ii) micropatterned substrates. Scale bar, 5 µm unless otherwise stated.

To determine the impact of controlled variations in surface microtopography on MSC morphology and function, micropatterned arrays were fabricated in a format that could easily be translated to standard cell processing techniques.^[^ ^40^ ^]^ To this end, UV nanoimprint lithography (NIL) was used to fabricate micro‐scaled features on OrmoComp, a hybrid organic–inorganic negative tone polymer. A total of three microstructure patterns and a single flat control (**Figure** **1**a) were developed in a single NIL step using OrmoComp on top of 13 mm *Ø* glass coverslips, enabling the assessment of four designs simultaneously. Using this format, we could fabricate highly reproducible structures over a wide surface area, suitable for accurate determination of the effects of the structures on a large number of cells. The fabricated structures exhibited grid patterns in which the spacing between micropillars was equal to the width of each pillar; being 1, 2.5, 5, 7.5, and 10 µm pillar widths with equal spacing (Figure 1b and Figure S1, Supporting Information), at heights of 1, 2.5, 5, 7.5, and 10 µm (Figure 1c). For the purpose of this study, grid designs are described in the format of pillar width and spacing × pillar height, so the 5 × 1 grid descriptor refers to 5 µm pillar width and spacing, and a height of 1 µm.

**Figure 2:**
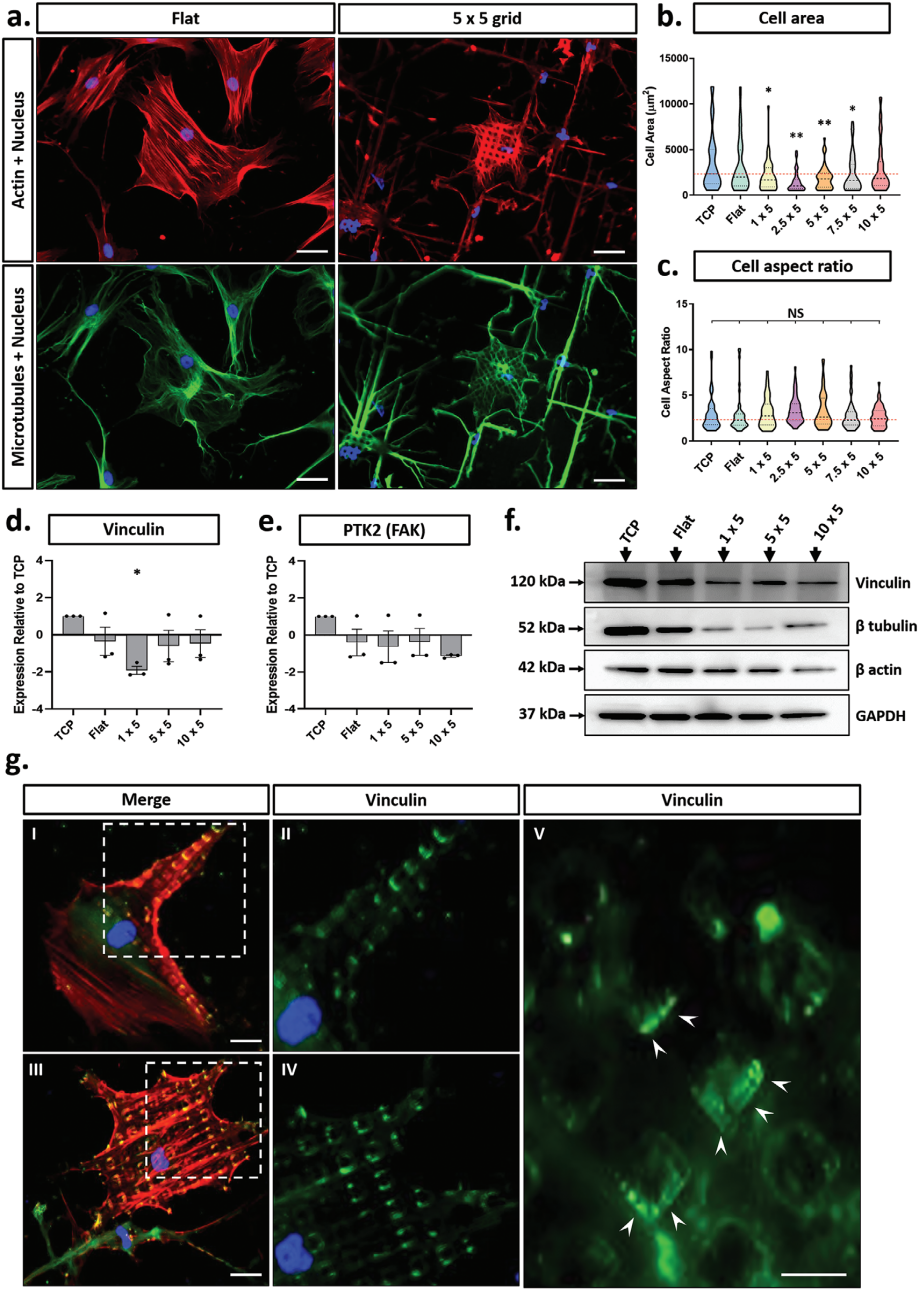
Characterization of MSC phenotype in response to substrate microtopography. a) Representative images of MSCs showing actin (red), microtubules (green), and nuclei (blue) on flat (control) and micropatterned substrates. Scale bar, 20 µm. b) Cell area and c) cell aspect ratio quantification on each micropattern design tested. d,e) Gene expression levels for vinculin and PTK2 (FAK) respectively as determined via RT‐PCR. f) Western blots of cytoskeletal and focal adhesion components in MSCs cultured on flat and micropatterned substrates. g) Representative immunofluorescent images of focal adhesions on 5 × 5 µm substrate designs, depicted with actin (red), vinculin (green), and nuclei (blue). Scale bars, 10 µm. All micropattern designs represent consistent micropillar heights of 5 µm. All graphs show mean ± SD for three independent MSC donors relative to control samples. Samples were analysed by one‐way ANOVA with Tukey post hoc testing. Statistically different samples are denoted by *p < 0.05, and **p < 0.005.

To confirm the ability of the OrmoComp substrates to support MSC adhesion and culture without any adverse effect on MSC viability, human bone marrow‐derived MSCs were seeded on all micropattern grid designs. SEM imaging and biocompatibility studies demonstrated efficient MSC adhesion and viability following 24 h culture (Figure S2a,b, Supporting Information), confirming a high degree of cytocompatibility with our micropatterned platform. On micropatterned substrates, cell adhesion primarily occurred on the top of the micropillars, with few cell‐substrate interactions in the spaces between each pillar (Figure 2d and Figure S3, Supporting Information). Interestingly however, there was evidence of decreased MSC metabolic activity and proliferation on micropatterned substrates compared to flat controls (Figure S2c,d,e, Supporting Information), providing a first indication that substrate topography may not only modulate cell attachment phenotypes, but also further influence cell behavior differently to that of flat substrates.

### 2.2 Interaction with Micropillars Modulates Cell and Nuclear Architecture

Having established a platform that enabled prolonged MSC adhesion on varying micropatterns, we next determined how changes in precisely ordered topographic features altered cell morphology. Immunostaining revealed that the cytoskeletal components *β*‐actin and *β*‐tubulin were clearly modulated by the specific geometry of the underlying substrate. MSCs cultured on the flat substrate displayed a random orientation of cell protrusions with no restriction on cell spreading, whereas the filipodia of MSCs cultured on the micropillars tracked along the *x* and *y* axes of the micropattern (Figure 2a and Figure S4a,b, Supporting Information). Quantification of morphological descriptors showed that cell spread area was significantly reduced for MSCs cultured on all micropatterned designs, as compared to flat controls, except for the 10 × 5 design. This effect was strongest for the 2.5 × 5 and 5 × 5 grid designs in which the cell area was decreased two‐fold compared to flat controls (Figure 2b). Conversely, cell aspect ratio showed negligible difference between cells cultured on micropatterned or flat control substrates (Figure 2c and Figure S4c, Supporting Information). Together these results suggest that specific micropatterned substrates modulate the direction of cell spreading, whilst decreasing the total cell area.

We next determined whether the observed changes in cytoskeletal organization were driven by alterations in the expression of key cytoskeletal proteins (Figure 2f and Figure S8b,c, Supporting Information). We observed a significant decrease in both *β*‐actin and *β*‐tubulin protein levels when compared directly to flat controls. This was especially pronounced for *β*‐actin which showed a 50% reduction in expression on the 1 × 5 and 5 × 5 grid designs, increasing to an approximate three‐fold reduction for the 10 × 5 designs. In contrast, lowest *β*‐tubulin expression was observed for cells on the 5 × 5 grid patterns with a four‐fold reduction in expression.

Given that the MSCs seemed to spread across the top surface of the micropillars, we investigated the effect this would have on the distribution of focal adhesions in the cell. Immunostaining for vinculin (a key component of focal adhesion complexes) revealed that focal adhesions formed exclusively on the top of the micropillars (Figure 2g) thereby meaning that the substrate architecture could be used to specify the spacing and distribution of FAs within the cell. Further quantitation of vinculin mRNA expression showed a significant decrease, compared to flat controls in only the 1 × 5 micropatterned design (Figure 2d). In contrast, paralleled Western blots indicated that vinculin protein levels were significantly reduced in MSCs on all micropatterned substrates when compared directly to flat controls (Figure 2f and Figure S8f, Supporting Information), with no significant variation observed between the 1 × 5, 5 × 5, and 10 × 5 designs. This discrepancy between mRNA and protein expression profiles suggests that regulation of vinculin occurs at the post‐translational level. qPCR for the focal adhesion marker, focal adhesion kinase (FAK), revealed no significant variation in expression between the different micropattern designs, or in comparison to flat control (Figure 2e).

Together these data show that MSC morphology is substantially influenced by the topography of the underlying substrate and that the specific effects are dependent upon the precise size of the features. MSCs spread across the top of the micropillars which restricts the cell spreading and shape, placing of focal adhesions, and ultimate cytoskeletal organization. These changes are further reflected by differences in the overall expression of major cytoskeletal and focal adhesion components. Our findings fit well with current suggestions that the cytoskeleton is stabilized by a tensile pre‐stress, generated by a complementary force balance between contractile actomyosin filaments and compression‐supporting microtubules.^[^ ^41^, ^42^, ^43^ ^]^

**Figure 3:**
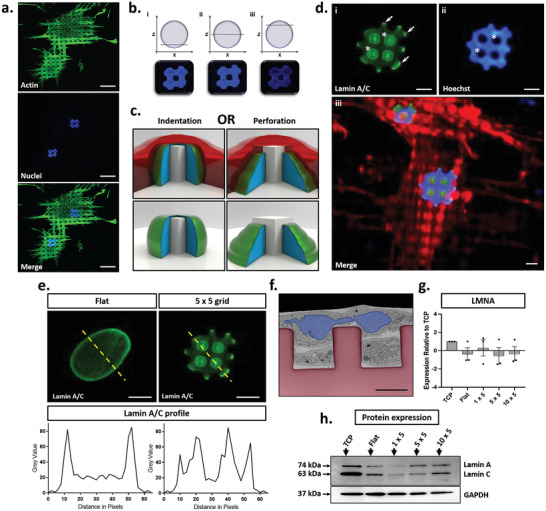
Characterization of nuclear indentation on micropatterned surfaces. a) Representative fluorescence microscopy images of MSC nuclear phenotypes on 5 × 5 micropatterned substrates, depicting actin (green) and nuclei (blue). Scale bar, 20 µm. b) Confocal z‐stacks demonstrating nuclear “hole” extending through the nuclear compartment. c) Schematic representation of two potential types of nuclear deformation, either nuclear indentation (in which the micropillar deforms the nuclear envelope, thus displacing genetic material), or nuclear perforation (in which a new nuclear envelope is formed around the micropillar to produce a doughnut shaped nucleus). Cytosol is shown in red, genetic material in blue, and nuclear envelope in green. d). Representative images of lamin A/C (green), DNA (blue), and actin (red). Asterisks demonstrate regions of lamin A/C accumulation on the surface of micropillars and corresponding regions of DNA displacement respectively. Arrows denote regions of peripheral nuclear envelope accumulation of lamin A/C. Scale bar, 5 µm. e) Quantification of lamin A/C staining on flat and the 5 × 5 substrates, respectively. f) Representative false‐coloured FIB/SEM image of MSCs cultured on 5 × 5 substrates. Micropillars are colored red, with nuclei colored blue. Scale bar, 5 µm g) RT‐PCR and h) Western blotting analysis of LMNA and lamin A/C expression profiles respectively across flat, 1 × 5, 5 × 5 and 10 × 5 micropillars. Data was collected from three independent replicates across three MSC donors. RT‐PCR data is presented as mean ± SD relative to TCP control samples.

In addition to the effects of micropatterns on cell morphology, Hoechst staining also showed distinct changes to the shape of nuclei in MSCs on the micropatterned surfaces **3a**. For some MSCs, this nuclear deformation was evident as an indentation on the periphery of the nucleus, whilst others appeared to have perforations or “holes” formed within the nucleus by the pillars. This was particularly evident in MSCs cultured on 5 × 5 grid patterns, in which we observed a high degree of “nuclear indentation” (Figure 3a). Upon closer inspection, the extent of this “indentation” was observed to pass through the entirety of the nuclear compartment, leading to a complete displacement of DNA around the micropillar itself (Figure 3b and Figure S5, Supporting Information). This observation correlates with other studies that have identified similar nuclear phenotypes,^[^ ^44^, ^45^, ^46^ ^]^ but further investigations into the nature and consequences of these deformations have not yet been performed. Given the emerging role of nuclear mechanotransduction and the role that forces play in shaping gene expression and subsequent cell fate, we aimed to further characterize both the structure and consequences of these altered nuclear phenotypes.

We determined that the nuclear phenotypes were caused by indentation of the nucleus, leading to complete displacement of the DNA around each micropillar. The integrity of the nuclear envelope (NE) was not affected and no perforation of the nuclear compartment took place (Figure 3c). Immunostaining for the inner nuclear membrane protein lamin A/C showed that even for areas of the nucleus where Hoechst staining was absent, lamin A/C staining was not diminished (Figure 3d). Cross‐sectional FIB‐SEM imaging further revealed a thin NE layer across the micropillar surface (Figure 3f, colour coded). Together, these results confirm that the integrity of the NE is maintained even when the micropillars cause significant rearrangement of the DNA within the nucleus.

As well as confirming that there was no perforation of the nuclear membrane, the immunostaining for lamin A/C in fact showed a distinct increase in intensity at the position of each of the micropillars. This contrasts with the cells on flat substrates in which lamin A/C staining was generally homogenous albeit for a brighter ring around the nuclear periphery (Figure 3e). Previous studies have suggested that accumulations of lamin A/C occur as a result of direct, localized tension across the NE.^[^ ^47^, ^48^ ^]^ Consistent with these reports, our staining localizes to regions that we hypothesize would be under the highest level of strain from cytoskeletal components. When combined with further observations of focal adhesion assembly appearing to grip micropillars surrounding the nucleus (Figure 2g), we propose that these nuclear deformations may arise from direct tension applied to the surface of the NE, driven by increased application of force from cytoskeletal tension, arising from micropillar‐induced nuclear deformation.

Further evaluation showed no significant differences in LMNA gene expression (which is the transcript for both lamin A and lamin C proteins) (Figure 3g) between TCP and flat controls, but levels of both lamin A and lamin C protein were reduced in MSCs on flat OrmoComp (Figure 3h and Figure S8d,e, Supporting Information). We believe this difference between TCP and flat OrmoComp may result from the inherent differences in the mechanical properties of the two materials which have a Young's modulus of ≈3 GPa and ≈1 GPa, respectively.^[^ ^19^ ^]^ Although we did not observe a significant variation in LMNA gene expression between flat and micropatterned conditions, lamin A and lamin C protein levels were significantly decreased in MSCs cultured on micropillars as compared to flat substrates (Figure 3h and Figure S8, Supporting Information). There were also variations between the specific micropatterns, with the lowest detected levels for both lamin A and C on the 1 × 5 grid design, followed by a gradual increase for 5 × 5 and 10 × 5 grids. Interestingly, the ratio of lamin A:C changed for MSCs on the 5 × 5 substrate (Figure S8h, Supporting Information). For flat, 1 × 5 and 10 × 5 substrates, the expression profiles of lamin A and lamin C remained the same with equal expression of both proteins, whereas on the 5 × 5, this ratio increased to 1.5, suggesting an increased proportion of lamin A in the NE. The ratio of lamin A:C has been demonstrated to safeguard nuclear architecture in response to direct force application across the NE^[^ ^49^ ^]^ and so we infer that our findings demonstrate a change in NE force transmission between micropatterned and flat control substrates. Thus, the modulation of both lamin A/C expression and localization supports the premise that the observed nuclear deformations are caused directly by tension generated across the NE.

### 2.3 Micropillar Dimensions Enable Selective Control of Nuclear Shape and Chromatin Remodeling

**Figure 4:**
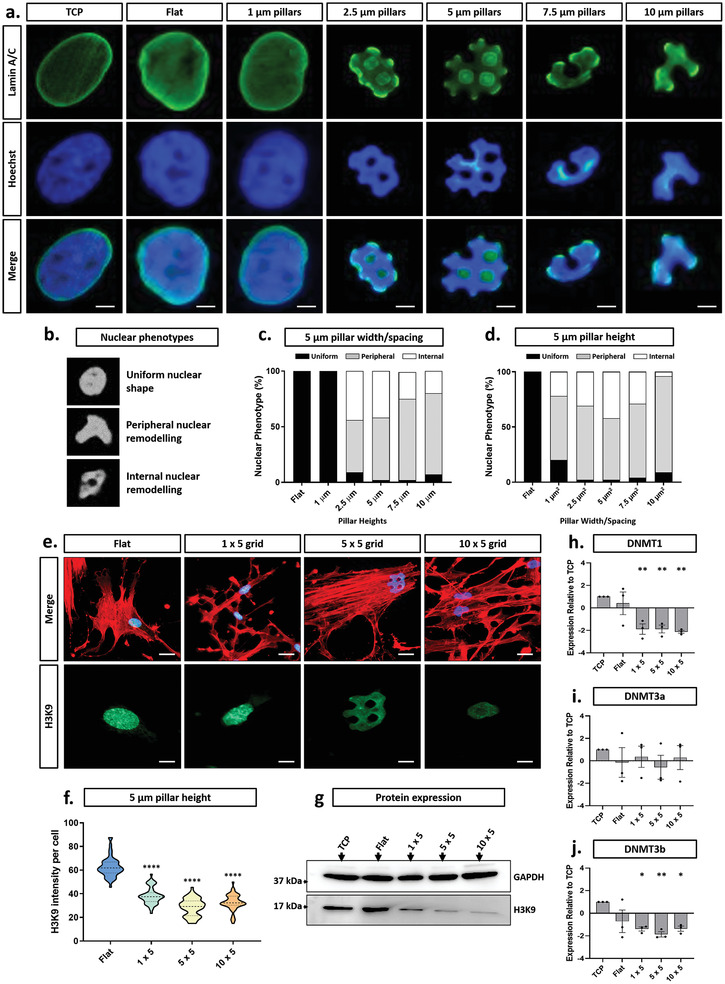
Micropattern‐defined nuclear indentation regulates heterochromatin expression and DNMT activity. a) Representative lamin A/C (green) and nuclei (blue) staining demonstrating phenotypes resulting from varied micropatterned spacing and width. Scale bar, 5 µm. b) Nuclear phenotype categories and associated quantification with changing c) micropillar height or d) micropillar width/spacing. e) Fluorescence staining of actin (red), nuclei (blue), and H3K9 (heterochromatin marker (green)). Scale bar, 5 µm. f) Mean fluorescent intensity quantification of H3K9 expression with changing micropillar width and spacing. g) Western blotting of H3K9 in MSCs cultured on control substrates and micropatterns with a constant height of 5 µm. h,i,j) RT‐PCR analysis of DNMT1, DNMT3a, and DNMT3b, respectively for each pattern at a constant 5 µm micropillar height. All graphs show mean ± SD for three independent MSC donors relative to TCP samples. Samples were analyzed by one‐way ANOVA with Tukey post hoc testing. Statistically different samples are denoted by *p < 0.05, **p < 0.01, and ****p < 0.001.

To better understand how micropillar geometry affected nuclear architecture, we analysed nuclear shape in MSCs on the complete range of micropattern designs (Figure 4 and Figure S9, Supporting Information). Immunostaining again showed that the micropillars were causing the cell nuclei to be deformed (Figure 4a). To quantify these effects, we developed a method for categorical shape assessment in which nuclear phenotypes were defined as “uniform”, having “peripheral remodeling” or “internal remodeling” (Figure 4b). Using this system of classification, it was evident that a micropillar height greater than 1 µm was required to initiate nuclear deformation (Figure 4c and Figure S9b–f, Supporting Information). Above this threshold, the nuclei of many cells were deformed with a varying degree of peripheral and internal remodeling. Of these, both 2.5 × 5 and 7.5 × 5 micropillars stimulated the greatest overall degree of deformation (>90%) whereas the greatest incidence of nuclear remodeling was observed for cells on the 5 × 5 micropillars, in which ≈45% of total nuclei had nuclear indentations, 50% had peripheral indentations, and only 5% had uniform nuclear architecture (Figure 4c,d). These findings correlate closely with current literature that also demonstrates that nuclei can be deformed by the topography of the underlying substrate.^[^ ^3^, ^4^, ^50^ ^]^ However, no other study has systematically categorized the effects of micropattern geometry on nuclear architecture and the nuclear envelope.

Since the regulation of chromatin organization is critical for cellular function, we aimed to determine whether these changes in overall nuclear structure affected chromatin organisation. To that end, immunostaining and Western blotting were used to determine whether there were changes to the distribution and overall amount of H3K9, a histone protein that marks heterochromatin. Methylation of H3K9 is a modification that is a well‐known indicator of silenced transcription and heterochromatin structure,^[^ ^51^ ^]^ and thus as our selected antibody specifically detects the tri‐methylated form of K9, we can gain an indication of chromatin organization in this context. For cells on flat surfaces, H3K9 was in a speckled distribution throughout the nucleus, but in the deformed nuclei of cells on micropillars, this speckled pattern was not evident and the overall fluorescent intensity was clearly attenuated (Figure 4e,f and Figure S10, Supporting Information). This correlated with the Western blotting data, which showed a trend of decreasing H3K9 as the pillar size/spacing increased, leading from a threefold reduction in total H3K9 in cells on the 1 × 5 micropillars to a fivefold decrease in cells on both the 5 × 5 and 10 × 5 designs (Figure 4g and Figure S8g, Supporting Information).

In order to confirm whether these changes in the level of heterochromatin would influence known gene regulatory factors, we analysed the expression profiles of three DNA methyltransferases, DNMT1, DNMT3a, and DNMT3b, each demonstrated to play unique and distinct roles in the maintenance of DNA methylation and thus gene activity.^[^ ^52^ ^]^ Since the most striking changes in nuclear architecture were identified at micropillar heights of 5 µm, we kept the micropillars at this constant height whilst changing the size/spacing of the pillars. Both DNMT1 and DNMT3b levels were reduced twofold when comparing micropatterned substrates to flat controls (Figure 4h,j) whilst no significant difference was observed for DNMT3a (Figure 4i). Interestingly, no significant differences were found between the different micropillar sizes, despite the stark differences in the degree of nuclear deformation.

Taken together, these results confirm that micropillars not only impact on the overall nuclear shape, but that their effects extend to the contents of the nucleus where they modulate both the chromatin and key regulators of genome architecture. This is perhaps not surprising given that much more subtle changes have previously been noted to control chromatin organization.^[^ ^53^, ^54^, ^55^ ^]^ The current literature assigns critical roles of DNMT1 and DNMT3b in the establishment of transcriptionally repressive chromatin during cell division.^[^ ^56^, ^57^ ^]^ Our observations therefore suggest that matrix topography plays an important role in the organization of the genome, which has the potential to impact upon gene expression. More specifically, the ability to control the degree of deformation of the nucleus by specifying the architecture of the underlying substrate may open new opportunities to regulate gene expression and subsequent cell fate.

### 2.4 Micropillar Dimensions Directly Influence Osteogenic Fate of MSCs via Modulation of Perceived Mechanical Properties

Given the significant impact of microtopography on both cell and nuclear morphology, we posit that the associated chromatin architectural changes arise from a change in the forces applied to the nuclear compartment, which in turn is directly determined by the changes to cytoskeletal arrangement. The microenvironmental regulation of tension within the cytoskeleton of MSCs is reportedly a key driving factor for mechanosensitive signaling and gene activity.^[^ ^58^ ^]^ Thus, we sought to further determine the extent of which our micropatterned platforms were able to modulate the osteogenic capacity of MSCs, and infer the precise mechanical cues driving this process.

**Figure 5:**
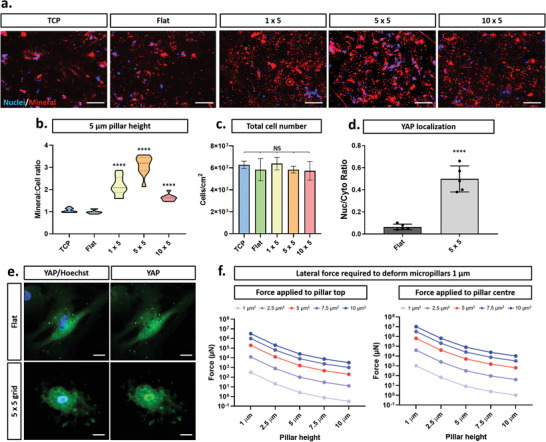
Micropatterned substrates enhance osteogenic capacity of MSCs. a) Representative mineralization images of MSCs, depicting nuclei (blue) and calcium deposition (red) on flat (control) and micropatterned substrates. Scale bar, 50 µm. b) Quantification of mineral generation per cell for 5 µm micropillar heights. c) Quantification of total cell number following 21 day osteogenic differentiation. d,e) Representative YAP fluorescence staining and quantification on flat and micropatterned substrates maintained at a constant micropillar height of 5 µm. Scale bar, 10 µm. f) Calculated force required to bend each micropillar by 1 µm, depicting forces applied at the top and middle of the micropillar. Graphs (b), (c), and (d) show mean ± SD from 300 cells for three hMSC donors. Samples were analyzed by one‐way ANOVA with Tukey post hoc testing. Statistically different samples are denoted by ****p < 0.001.

MSCs were cultured on micropillars of varying sizes in the presence of osteogenic supplements and the extent of mineralization determined after 21 days. Significant increases in mineral deposition were observed across all micropatterned substrates as compared to flat controls (Figure 5a and Figure S11, Supporting Information). Subsequent quantification of the mean fluorescent intensity revealed that three times as much mineral/cell was produced for MSCs on the 5 × 5 micropillars, and twice as much on the 1 × 5 and 10 × 5 micropillars as compared to flat controls (Figure 5b and Figure S11, Supporting Information). These differences were observed to occur despite minimal differences in total cell number between conditions following 21 days osteogenic differentiation (Figure 5c).

Given that MSCs are highly mechanosensitive^[^ ^17^, ^58^, ^59^ ^]^ and that we had described significant changes to cytoskeletal arrangement, focal adhesion formation, and nuclear phenotypes, we hypothesized that the large increase in osteogenic capacity of MSCs on the 5 × 5 design was directly caused by the changes in cellular architecture and mechanotransduction. To confirm this hypothesis, immunostaining of the mechanosensitive transcription factor YAP (Yes Associated Protein) was conducted. YAP shuttles between the nucleus (where it is active) and cytoplasm depending upon mechanical cues^[^ ^60^ ^]^ and the nuclear localization of YAP has been shown to promote MSC osteogenesis.^[^ ^61^ ^]^ Our results showed 89% of cells have predominantly cytoplasmic YAP on flat substrates (Figure 5d,e), which interestingly contradicts previous reports for MSCs on substrates of a high modulus.^[^ ^62^ ^]^ In stark contrast, YAP was present in the nucleus of 48% of cells on the 5 × 5 designs (the conditions under which the osteogenic response was the strongest). This increased accumulation of YAP in the nuclear compartment of MSCs cultured on micropillars shows a direct influence of topographic cues on MSC mechanotransductory machinery. Given the positive role that nuclear localization of YAP has upon MSC osteogenesis, this may also contribute to the enhanced osteogenic capacity of MSCs on the micropatterned substrates. In contrast, it was previously demonstrated that silica nanoneedles were shown to decrease nuclear accumulation of YAP when compared to flat controls.^[^ ^63^ ^]^ We believe that these observed differences are attributed to the varied cellular responses between nano‐ and micro‐scaled topographical cues. The “sharp” nature of structures used by Hansel et al. may also be a key contributor to this difference, as we observe deformation of the nucleus across the structure over tens of microns whereas the previous study shows distinct foci of acute tension from the tip of the needle.

To determine whether the change in YAP localization was a consequence of the topography of the micropatterned substrates or caused by perceived changes in the stiffness of the substrate due to variations in micropillar bending (dependent on aspect ratio) as reported previously,^[^ ^64^ ^]^ we conducted modeling to predict the required force to deform each pillar design tested (Figure 5f). The force required to deform even the weakest micropillar structure by 1 µm (1 × 10) is 318 nN, which greatly exceeds the force that a cell can exert on a substrate (≈40 nN).^[^ ^65^ ^]^ We therefore conclude that the enhanced osteogenic capacity of MSCs on the 5 × 5 design is induced by a specific response to the topography of the substrates and not by perceived changes to the substrate mechanical properties caused by micropillar deformation. We can therefore determine that the occurrence of pillar deformation as observed in Figure S3, Supporting Information, is likely an artefact from cryo‐processing for SEM. When combined with our observations thus far, these results indicate that the enhanced osteogenic capacity of MSCs originates from the modulation of cellular force, defined by changing focal adhesion composition, cytoskeletal organisation, and mechanosensitive transcription factor regulation determined by select micropillar aspect ratios and spacing. This is consistent with previous studies into the selective localization of ligand spacing,^[^ ^66^, ^67^, ^68^ ^]^ demonstrating that MSC lineage specification can be regulated by precise modulation of spacing between cell attachment sites.

### 2.5 Enhanced MSC Osteogenesis on Micropillars is Caused by Modulation of Myosin II‐Regulated Cytoskeletal Tension

**Figure 6:**
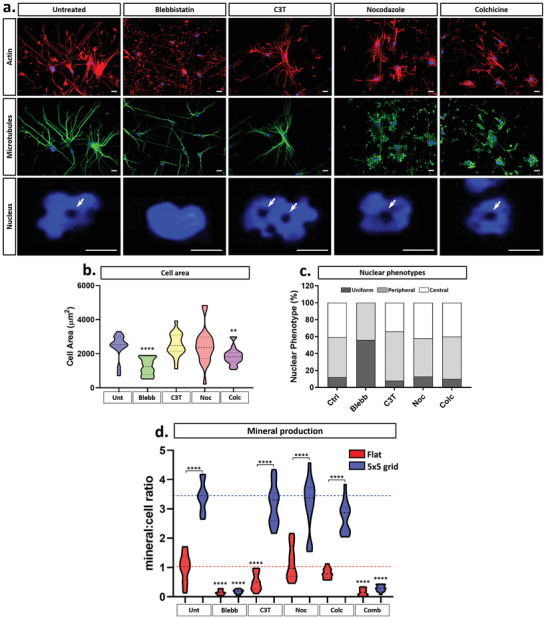
Nuclear indentations and associated enhanced osteogenic capacity of MSCs are determined by myosin II‐dependent actin contractility. a) Representative fluorescence microscopy images of MSCs treated with actin and microtubule inhibitory drugs during culture on the 5 × 5 micropatterned design, with actin (red), microtubules (green), and nuclei (blue). Arrows denote regions of nuclear indentation and DNA displacement around the micropillars. Scale bar, 10 µm. b) Cell area and c) associated nuclear phenotype quantification of MSCs following drug treatment on the 5 × 5 micropillars. d) Quantification of mineralization at 21 days of osteogenesis in MSCs treated with actin and microtubule inhibitory drugs during culture on the control and 5 × 5 micropatterned design. All graphs show mean ± SD from across 300 cells for three independent MSC donors relative to control samples. Samples were analyzed by one‐way ANOVA with Tukey post hoc testing for comparisons within either Flat or 5 × 5 conditions. For comparisons between flat and 5 × 5 conditions, *t*‐tests were performed. Statistically different samples are denoted by **p < 0.01, ****p < 0.001.

One model to explain our data is that the micropillars drive changes to the MSC cytoskeleton and nucleus, which in turn alters chromatin structure and ultimately influences the osteogenic differentiation potential of the cells. To test this hypothesis, we used a range of drugs to disrupt actin (blebbistatin (myosin II inhibitor) and C3T (RhoA inhibitor)) or microtubule (nocodazole (polymerisation inhibitor) and colchicine (anti‐mitotic inhibitor)) function in MSCs on both flat and 5 × 5 substrates (Figure 6a and Figure S12, Supporting Information) which present both the lowest and highest degree of osteogenesis respectively (Figure 5a,b). As expected, on the flat substrates, blebbistatin significantly disrupted actin arrangement, whereas C3T caused a more subtle change in cell morphology. The addition of both microtubule destabilising drugs significantly altered microtubule organisation. Strikingly, the central nuclear deformations seen in MSCs on 5 × 5 micropillars were retained under all conditions tested other than those treated with blebbistatin. Blebbistatin caused a significant reduction in total cell area (Figure 6b) and stimulated the total loss of central nuclear deformations (Figure 6c). Blebbistatin specifically targets actin contractility by directly inhibiting myosin II ATPase activity and subsequent actomyosin contractility. Thus, we conclude that the nuclear “indentations” in MSCs on the 5 × 5 design were caused by the application of tension by the cytoskeleton, specifically the contractility of actin filaments and not the organization/formation of new actin or the microtubule network.

The presence of the nuclear changes is directly linked to the enhanced osteogenic response of the MSCs on the 5 × 5 design as seen in Figure 6d, in which the osteogenic differentiation of MSCs on the flat control and the 5 × 5 design with the addition of the cytoskeletal inhibitors both individually and in combination is presented. Quantification of the mineralization at 21 days indicated that it was decreased fourfold by blebbistatin treatment in MSCs on flat substrates. However, on the 5 × 5 micropillars, this decrease was significantly higher at 15‐fold lower than untreated controls. These findings confirm that actomyosin contractility is necessary for MSC osteogenesis and further demonstrate that the enhanced osteogenic capacity of MSCs on the 5 × 5 design requires force generation across the NE to be maintained, thereby defining a new role for nuclear force transmission in MSC fate determination. Furthermore, in accordance with our previous work,^[^ ^58^ ^]^ the addition of C3T (a RhoA inhibitor) decreased mineral deposition in MSCs on flat substrates. However, in cells on the 5 × 5 design, mineral deposition was unchanged from that of untreated controls. Our results demonstrate that the precise reorganization of nuclear architecture on the 5 × 5 design mitigates the action of C3T inhibition of RhoA activity, either by diminishing the effect of drug action, or by the stimulating osteogenesis via alternative mechanisms that counteract the effect of the RhoA inhibition.

Overall, we have demonstrated that micropattern geometries modulate nuclear architecture changes via direct tension across the NE, which in turn regulates chromatin organization and subsequent gene activity. Our systematic application of precise microscale adjustments to pillar heights and spacing have demonstrated the importance of changes on this size scale in modulating cell fate. Our findings further elucidate an optimal type of nuclear deformation enabled by the 5 × 5 grid design, which provides sufficient changes to internal nuclear organization to enable the activation of mechanotransductory signaling pathways, similar to that observed in the MSC response to changing substrate stiffness.^[^ ^11^, ^17^, ^58^ ^]^ The identification of this novel signaling mechanism highlights the potential of micropatterned biomaterials as tools for both regenerative medicine and clinical application. This has an advantage over the use of chemical inhibitors, growth factors, and small molecules, because topographic patterning of substrates is non‐invasive and does not require the use of non‐physiological chemicals to modulate cellular function. However, microstructured materials are currently limited by the scale of replication, the size of the surface area available for cell interactions (which is typically small), and the transition of microtopographies from 2D substrates to more physiologically relevant 3D structures. Therefore, increasing our understanding of the mechanotransductory mechanisms which underpin these cellular effects remains key to engendering future applications of microtopographies in clinical settings.

## 3 Conclusion

In this study, we generated a library of precise microstructures on OrmoComp using UV nanoimprint lithography. The quality and reproducibility of these structures across a relatively large scale enabled systematic investigation of the effects of micropillar size and height on cell morphology, mechanotransduction, and fate. Our data provide fundamental insights into the underlying mechanisms of mechanotransduction, showing that nuclear and chromatin architecture is regulated by cytoskeletal tension, which in turn is influenced by specific microtopographies. This interplay has subsequent effects on cell fate. We demonstrate that MSC osteogenesis is enhanced by specific micropillar designs that alter chromatin organisation and gene expression, which are directly related to the nuclear deformation caused by myosin‐II‐generated tension on the actin cytoskeleton. Interestingly, osteogenesis on micropatterned (but not flat) substrates was enhanced, even in the presence of RhoA inhibitors, revealing the role of substrate topography in regulating actin turnover and associated cellular tension. Although many micropatterns enhanced MSC osteogenesis, a significant increase was shown above all others for 5 × 5 micropillars; these findings are intriguing since they identify a highly specific micropillar size/spacing and height for optimal modulation of nuclear indentation and associated cellular function. Together these findings advance fundamental understanding of mechanotransduction, showing a new role for substrate microtopography in directing force transmission across the nucleus to modulate chromatin organisation, gene expression, and cell fate. Harnessing surface microtopography instead of biological factor supplementation to direct cell fate has far‐reaching ramifications for smart cell cultureware in stem cell technologies and cell therapy, as well as for the design of smart implant materials with enhanced osteoinductive capacity.

## 4 Experimental Section

##### Substrate Fabrication and Functionalization

The fabrication of micropatterned substrates by means of UV nanoimprint lithography started with the fabrication of corresponding master molds. UV lithography using SU‐8 was used to create the master molds with resolutions of 2.5 µm and higher. To fabricate 2.5–10 µm height structures, SU‐8 was spin coated onto silicon wafers at 3000 rpm for 40 s, then subsequently baked at 95 °C for 180 s. UV exposure was then conducted (365 nm, 14 W) for 8 s, followed by a further baking step at 90 °C for 1 min and a final developing step at 45 s to reveal the final structures.

To fabricate the 1 µm × 1 µm pattern design, electron beam lithography (EBL) was used. Silicon was first spin‐coated with poly(methyl methacrylate) (PMMA, 950 K A4) at 4250 rpm for 45 s to generate a 150 nm thick layer. Exposure was then conducted using the electron beam (Vistec EBPG 5000+ESHR) operating at 100 kV, using an optimized dose of 800 µC cm^−2^, followed by development in a methyl–isobutyl–ketone and isopropanol mixture. The resulting resist was then used in a lift‐off process to create a chromium structure which in turn was used as a mask in deep reactive ion etching (DRIE) of the substrate in SF6/C4F8 gases standard Bosch process using (Oxford Instruments PlasmaLab 100 ICP380).

Each master mold was then silanized with trichloro (1H,1H,2H,2H‐perfluorooctyl) silane, by chemical vapor deposition to minimize the adhesion of the cast PDMS. Microstructures on these molds were then transferred onto PDMS substrates via soft lithography using Sylgard 184 Silicone Elastomer. In brief, base and curing agents (10:1 by weight) were mixed thoroughly and then kept under vacuum (2 × 10^−1^ mbar) for 20 min to remove all entrapped air. The mixture was then cured at 65 °C for 2 h.

UV‐curable inorganic–organic hybrid polymer OrmoComp was used for UV‐NIL. Briefly, OrmoComp droplets were dispensed onto O_2_ plasma treated glass substrates, followed by UV‐NIL using an EVG 6200 mask aligner (365 nm, 14 W) for 25 s to produce the final micropatterns of OrmoComp on the glass substrate. Following fabrication, sample specifications were validated using SEM (as defined in Scanning Electron Microscopy in the Experimental Section) and optical profilometry using a Bruker Contour GT‐I 3D‐optical microscope.

##### Mesenchymal Stem/Stromal Cell (MSC) Culture

Human Bone marrow derived MSCs were cultured in DMEM‐low glucose supplemented with 100 U per mL penicillin, 100 µg mL^−1^ streptomycin (DMEM/PS) and 10% fetal bovine serum (FBS) at 37 °C and 5% CO_2_. These MSCs are tested and certified to meet the required ISCT defined criteria for stem cells and are free from tested pathogens. All cultures were routinely tested for mycoplasma every 3 months. Prior to all experiments, cells were serum‐starved overnight in DMEM/PS with 0.25% FBS. All experiments were conducted using passage 6 cells. Drug Inhibitors were added to cell cultures at the following concentrations: blebbistatin (myosin inhibitor, 50 µm), C3T (RhoA inhibitor, 1 µg mL^−1^), colchicine (microtubule polymerization inhibitor, 5 µg mL^−1^), and nocodazole (microtubule polymerization inhibitor, 5 µm).

##### Osteogenic Differentiation

MSCs were plated onto micropatterned substrates at a density of 5 × 10^3^ cells cm^−2^. Following 24 h, cells were treated with osteogenic inductive medium (DMEM low‐glucose with 10% FBS, 50 µm ascorbate‐2‐phosphate, 100 ng mL^−1^ dexamethasone, and 10 mm *β*‐glycerophosphate), with medium changes performed every 3 to 4 days. At 21 days, differentiation was quantified using xylenol orange (Sigma) and OsteoImage (Lonza) staining assays to detect mineral deposition.

For OsteoImage mineralization assays, samples were processed as per the manufacturer instructions following 15 min fixation in 4% paraformaldehyde (PFA) diluted in phosphate‐buffered saline (PBS). For xylenol orange assays, samples were washed with PBS and incubated in PBS containing Hoechst 33 342 [1:1000] for 20 min. Samples were then rinsed thrice in PBS, followed by incubation in xylenol orange working solution (20 µm xylenol orange in dH_2_O) for 30 min. Images were collected using a Nikon Eclipse Ti microscope equipped with Spot imaging software.

Mineral quantification was performed using ImageJ to calculate corrected total cell fluorescence (CTCF) using the intensity density function. Detected fluorescence intensity/unit area was normalized to cell number as determined by nuclear staining, providing a value representing mineral deposition per cell. To ensure systematic and non‐biased analysis of the data sets, excitation wavelengths and powers were kept consistent between samples, with image acquisition conducted in identical regions.

##### Quantitative Real‐Time RT‐PCR for mRNA

To analyse mRNA, total RNA was extracted using the Qiagen RNeasy mini kit with on‐column DNase treatment according to the manufacturer's instructions. cDNA was synthesized from up to 750 ng RNA using Superscript VILO in a total volume of 20 µL. Reverse transcription was performed in the Biorad T100 Thermal Cycler using the following cycling conditions: 10 min at 25 °C, 60 min at 42 °C, and 5 min at 85°C. Quantitative PCR reactions were set‐up in a total volume of 10 µL with 1 × ABI Fast SYBR Green Mastermix and 0.2 µm forward and reverse primers. The primer sequences were listed in Table S1, Supporting Information. A CFX96 Real‐Time System (Bio‐Rad) was used to run the samples with fast cycling parameters of 20 s at 95 °C, 3 s at 95°C, and 30 s at 60°C, which was repeated for 40 cycles and followed by a melt curve. Data were analyzed by the 2^−ΔΔ^Ct method using RPS27a as a reference gene.

##### Western Blotting

Cell lysates were prepared in RIPA buffer, supplemented with protease inhibitor cocktail [1%]. Lysates were subsequently combined with 5 × Laemmli sample buffer and processed through SDS‐PAM gel electrophoresis (SDS‐PAGE) using pre‐cast 4–12% gradient gels and subsequent Western blot analysis. Following electrophoresis, proteins were transferred to PVDF membrane under wet electro‐transfer conditions using transfer buffer (ethanol [10%], Tris [25 mm], SDS [0.1%], and glycine [190 mm] in H_2_O) maintained at 350 mA for 1 h (at 4 °C). Membranes were subsequently blocked in PBS and 5% skimmed milk powder at room temperature (RT) for 30 min and incubated overnight in primary antibody (diluted in blocking solution) at 4 °C; *β*‐tubulin [1:1000] (Sigma T8328), GAPDH [1:2000] (Millipore 6C5), *β*‐actin [1:1000] (Sigma A5316), lamin A/C (Jol2) [1:500] (ImmuQuest IQ608), H3K9 [1:1000] (Abcam 176 916), vinculin [1:1000] (Sigma V9131). Membranes were incubated in secondary antibody for 1 h at RT; goat anti‐rabbit conjugated POD [1:3000] (Abcam ab6721), goat anti‐mouse conjugated POD [1:4000] (Abcam ab6728). Chemiluminescence signal was detected using Pierce ECL Plus solution and a UVITEC mini HD6 detector.

##### Scanning Electron Microscopy (SEM)

Following MSC culture on micropatterned substrates, samples were incubated in BRB80 buffer (PIPES [80 mm], MgCl_2_ [1 mm], EGTA [1 mm], pH 6.8 with KOH) + digitonin [0.001%] for 10 min. Fixation was performed for 2 h in PHEM buffer (HEPES [5 mm], PIPES [60 mm], EGTA [10 mm], MgCl2 [2 mm], pH 7 with KOH) supplemented with glutaraldehyde [2%] and tannic acid [0.1%]. Samples were subsequently incubated in Osmium tetroxide 0.1% for 30 min, dehydrated through 5 min washes in 10%, 20%, 40%, 60%, and 80% ethanol and washed twice for 5 min in 100% ethanol. Critical point drying was then performed using a Balzers CPD 030, followed by sputter coating with a 10 nm platinum layer using a 328 Cressington sputter coater. Samples were imaged using a Hitachi S5200 scanning electron microscope.

##### Focused Ion Beam Scanning Electron Microscopy (FIB‐SEM)

Samples were rinsed with 0.1 m sodium cacodylate buffer and fixed in 2.5% glutaraldehyde diluted in the same buffer at 4 ˚C overnight. Following this, samples were washed (3 × 5 min) with chilled 0.1 m sodium cacodylate buffer and quenched with chilled 20 mm glycine solution diluted in the same buffer for 20 min. After repeating the washing step, samples were post‐fixed by combining equal volumes of 4% aqueous osmium tetroxide with 2% potassium ferrocyanide in 0.2 m sodium cacodylate buffer on ice for 1 h. Samples were then washed again (3 × 5 min) with chilled buffer and incubated with 1% tannic acid (BDH) in water at RT for 20 min. After rinsing with buffer (2 × 5 min), samples were further incubated with 2% aqueous osmium tetroxide at RT for 30 min. Following this, samples were washed (2 × 5 min) with distilled water and incubated with syringe‐filtered 4% aqueous uranyl acetate (UNIVAR) at 4 ˚C overnight. Samples were then washed (3 × 5 min) with chilled distilled water and gradually dehydrated with increasing concentrations of ethanol; 10%, 30%, 50%, 70%, 90%, and 100% (1 × 7 min) at RT. 20 mL Epon 812 resin was prepared by initially mixing 12.2 g of DDSA (Dodecenyl Succinic Anhydride Specially Distilled 13 710, Electron Microscopy Sciences), 4.4 g of Araldite (GY 502 10 900, Electron Microscopy Sciences), and 6.2 g of Procure 812 (EMBED 812 RESIN 14 900) using a mechanical stirrer. Once the solution was uniformly mixed, 0.8 mL of BDMA (*N*‐benzyldimethylamine 11 400, Electron Microscopy Sciences) was added to it while stirring. Samples were then infiltrated with increasing concentrations of the freshly prepared resin solution in 100% ethanol at RT and in a sealed container using the following ratios: 1:3 (3 h), 1:2 (3 h), 1:1 (overnight), 2:1 (3 h), 3:1 (3 h). Following this, samples were finally infiltrated with 100% resin solution overnight. Prior to polymerization at 60 ˚C, the excess resin was drained away by mounting the samples vertically for 1 h.

To image, samples were mounted into FIB‐SEM stubs and sputter coated with gold and sectioned using a Thermo Fischer Helios Nanolab 600 FIB‐SEM. Regions of interest were protected from ion beam (i‐beam) damage using i‐beam assisted deposition of a ≈0.6 µm thick platinum layer. The coating was carried out at 30 kV using i‐beam current of 0.92 nA. Following this, rough milling was performed at acceleration voltage of 30 kV and a current ranging between 6.5–9.3 nA. The resulting cross sections were then polished with a voltage of 30 kV and a current of 2.8 nA. Images were taken using an electron beam at acceleration voltage of 2 kV, a current of 0.34 nA using immersion mode, and with a TLD detector operating in back‐scattered (BS) electron collection mode, at a dwell time of 100 µs. Images were black‐white inverted.

##### Immunofluorescence Staining

Cells grown on micropatterned substrates were fixed in 4% PFA diluted in PBS for 15 min followed by permeabilization with 0.5% Triton X‐100 for 10 min. Fixed samples were blocked in 5% bovine serum albumen (BSA) for 30 min and incubated with primary antibodies (diluted in BSA) for 1 h at RT. The following antibodies were used throughout this investigation: *β*‐actin [1:1000] (Sigma A5316), *β*‐tubulin [1:1000] (Sigma T8328), lamin A/C (Jol2) [1:500] (ImmuQuest IQ608), H3K9 [1:1000] (Abcam 176 916), and vinculin [1:1000] (Sigma V9131). Cells were extensively washed in PBS and then incubated with the relevant secondary antibodies for 1 h, which were conjugated with Alexa Fluor 488 or Alexa Fluor 555. DNA was counterstained with Hoechst 33 342 [1:1000] for 20 min. All samples were analysed by either conventional fluorescence microscopy using a Nikon Eclipse Ti microscope, or confocal laser‐scanning microscopy using either a TCS‐SP1/SP5 (Leica) or an A1 HD25 (Nikon) microscope.

##### Biocompatibility Screening

To ensure cell phenotype changes did not arise from negative responses to the base OrmoComp substrate, biocompatibility was tested as described previously.^[^ ^69^ ^]^ Briefly, cell viability was assessed using Live/Dead staining assay (ThermoFisher) following manufacturers protocol. Proliferation was monitored using Ki67 fluorescence staining as detailed in Immunofluorescence Staining in the Experimental Section. Finally, changes to metabolic activity were probed using the MTS assay (Promega) following manufacturers protocol. Each viability assay was conducted 72h post cell seeding.

##### Force Modeling

Lateral force required to deform the micropillars by 1 µm was calculated by modeling each pillar as a squared cantilever and assessing the stiffness required to bend the cantilever at a given position along their height. Stiffness (*k*) of a square cantilever was calculated as:

(1) $$k=\frac{Ya^{4}}{2p^{2}\left(3h-p\right)}\;$$

Where *a* is the lateral size of the pillar, *h* is the height of the pillar, *p* is the position at which the force is applied, being 0 at the surface and equal to h at the top, and *Y* is the Young's modulus of the pillar (1.27 GPa).^[^ ^70^ ^]^

##### Drug Inhibition

For screening of morphological changes in MSCs resulting from inhibitor addition, cells cultured on micropatterned substrates were seeded at a density of 5 × 10^3^ cells cm^−2^. After 24 h to allow cell attachment, inhibitors were added to cell cultures at the following concentrations: blebbistatin (myosin inhibitor, 50 µm, Merck), C3T (RhoA inhibitor, 1 µg mL^−1^, Cytoskeleton Inc.), colchicine (microtubule polymerization inhibitor, 5 µg mL^−1^, Merck), and nocodazole (microtubule polymerization inhibitor, 5 µm, Merck) and incubated for 4 h, following which cells were fixed and assessed as described in Immunofluorescence Staining in the Experimental Section.

For extended differentiations supplemented with inhibitors, cells were prepared as described in the osteogenic differentiation section, with inhibitors added directly to osteogenic media. Media changes were performed every 3–4 days.

##### Statistical Analysis

All graphical data is presented as mean ± standard deviation across 3 separate MSC donors (*n* = 9) unless otherwise stated. A Kolmogorov–Smirnov test was used to test data for Normal distribution and Levene's test used to determine homogeneity of variance. Data with a Normal distribution were analysed by one‐way ANOVA and Tukey (equal variance) or Games–Howell (unequal variance) post hoc tests. Non‐parametric data were analysed by Kruskal–Wallis test. All statistical analyses was performed using GraphPad Prism 8.

## Conflict of Interest

The authors declare no conflict of interest.

## Supporting information

Supporting information
