# Supplementary material for: Precision Surface Microtopography Regulates Cell Fate via Changes to Actomyosin Contractility and Nuclear Architecture
Source: Adv Sci (Weinh). 2021 Jan 29;8(6):2003186. doi: 10.1002/advs.202003186 (PMC7967085; doi:10.1002/advs.202003186)
Supplement: Supplementary file 1 — Supporting information [file ADVS-8-2003186-s001.pdf]

© 2020 Wiley-VCH GmbH

ADVANCED SCIENCE
Open Access

Supporting Information

for *Adv. Sci.,* DOI: 10.1002/advs.202003186

Precision surface microtopography regulates cell fate via changes 1 to actomyosin contractility and nuclear architecture

J. Carthew, H.H. Abdelmaksoud, M. Hodgson-Garms, S. Aslanoglou, R. Elnathan, J. P. Spatz, J. Brugger, H. Thissen, N. H. Voelcker, V.J. Cadarso*, J. E. Frith**

Precision surface microtopography regulates cell fate via changes to actomyosin contractility and nuclear architecture

J. Carthew[1,2], H.H. Abdelmaksoud[3,4], M. Hodgson-Garms[1], S. Aslanoglou[4,5,10], R. Elnathan[1,4,5], J.P. Spatz[6,7,8], J. Brugger[9], H. Thissen[10], N.H. Voelcker[1,4,5,10*], V.J. Cadarso[2,3,4*], J.E. Frith[1*].

Supplementary Figures

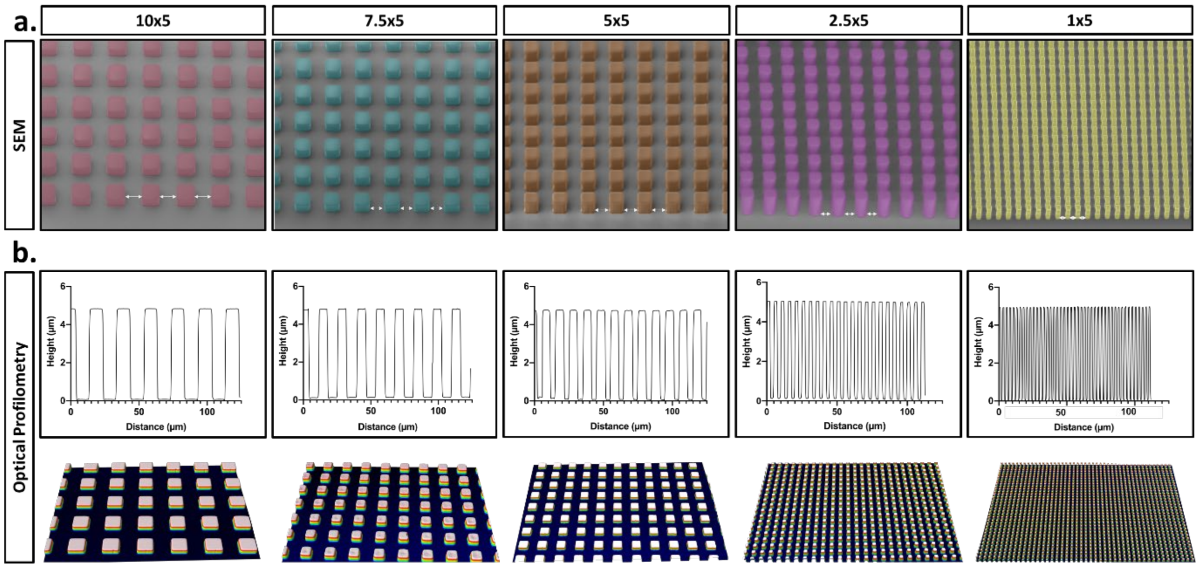

Figure S1. (a) False coloured SEM images of micropatterned substrates demonstrating how dimensions were calculated using ImageJ. Arrows depict measurement spacing between each micropillar. (b) Optical profilometry measurements to ensure pillar diameter is consistent between surface and base regions.

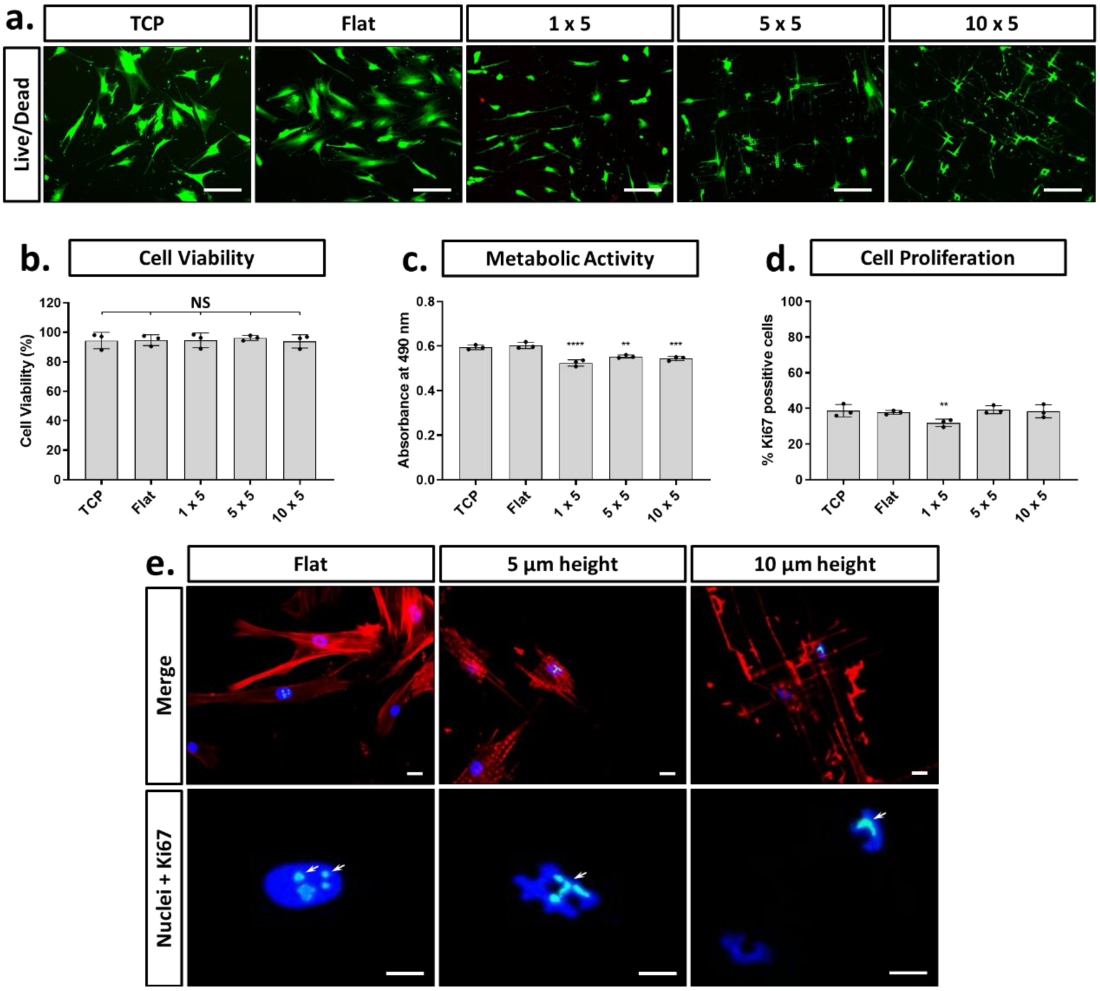

Figure S2. MSC culture on micropatterned substrates decreases metabolic activity, whilst retaining comparable viability and proliferation capacity to flat controls. (a). Representative Live/Dead staining (green/red) of MSCs cultured on flat (control) and 5 µm high micropillars. Scale bars, 50 µm. (b), (c) and (d) show cell viability, metabolic activity and proliferation respectively. (e) Representative ki67 quantification and staining on flat and 5 µm high micropillars. Scale bar, 7.5 µm. All graphs show mean ± SD for three independent MSC donors relative to flat control samples. Samples were analysed by one-way ANOVA with Tukey post hoc testing. Statistically different samples are denoted by $^{**}p < 0.01$, $^{***}p < 0.005$, and $^{****}p < 0.001$.

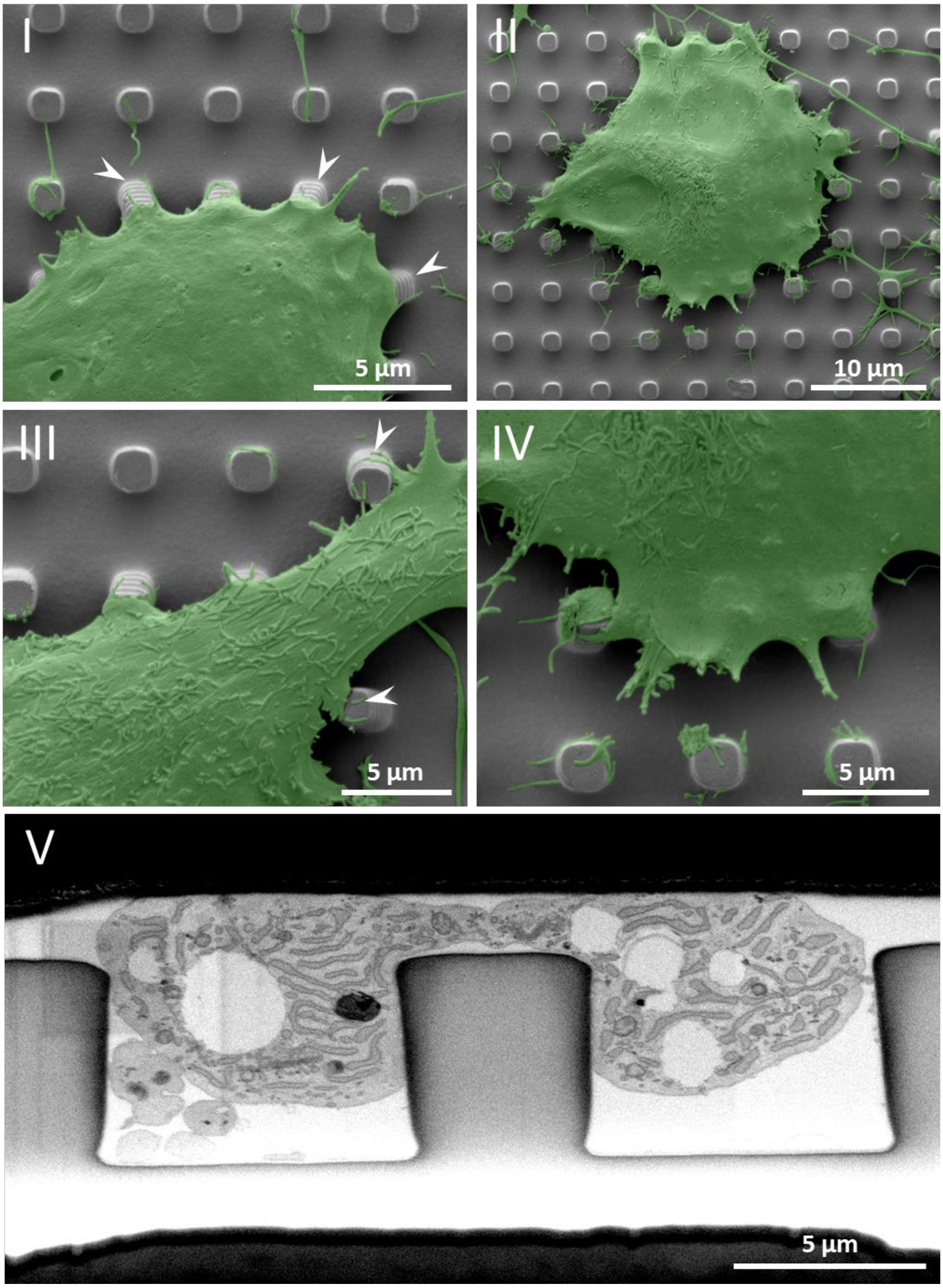

Figure S3. (I-IV) False coloured SEM images of MSCs cultured on micropatterned substrates demonstrating cell-substrate interactions. Arrows depict micropillar bending, which we suggest is a result of SEM processing due to our modelling presented in figure 5f. (V) FIB SEM imaging validating that micropillars are not bending due to cellular traction force.

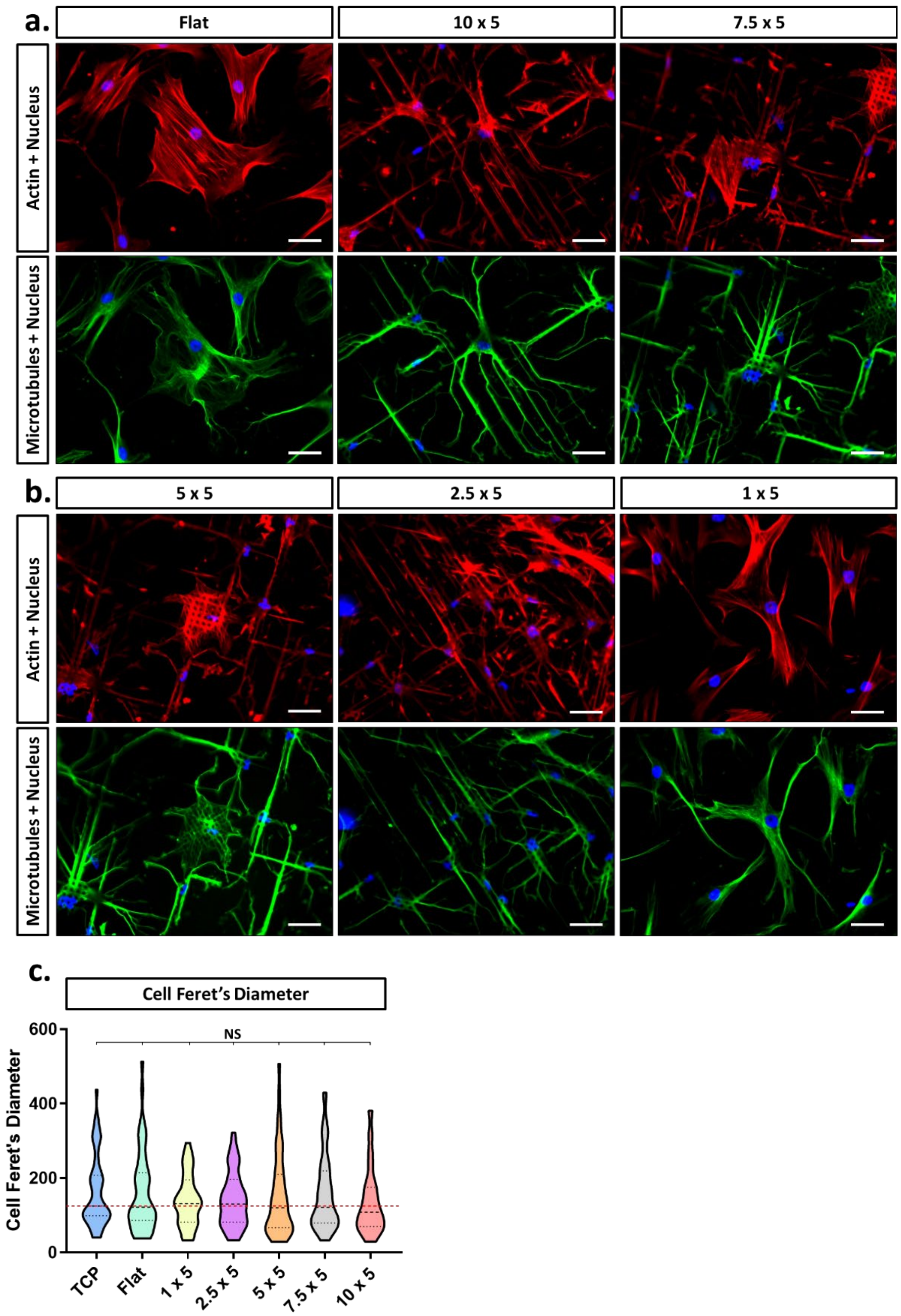

Figure S4. MSC morphology is dependent on the precise dimensions of micropatterned substrates. (a) and (b) denote actin (red), nuclear (blue) and microtubule (green) staining of MSCs cultured on 5 µm high micropatterns with varied width and spacing. Scale bars, 20 µm. (c). Quantification of cell feret's diameter for MSCs cultured on micropillars. All graphs show mean ± SD for three independent MSC donors relative to control samples.

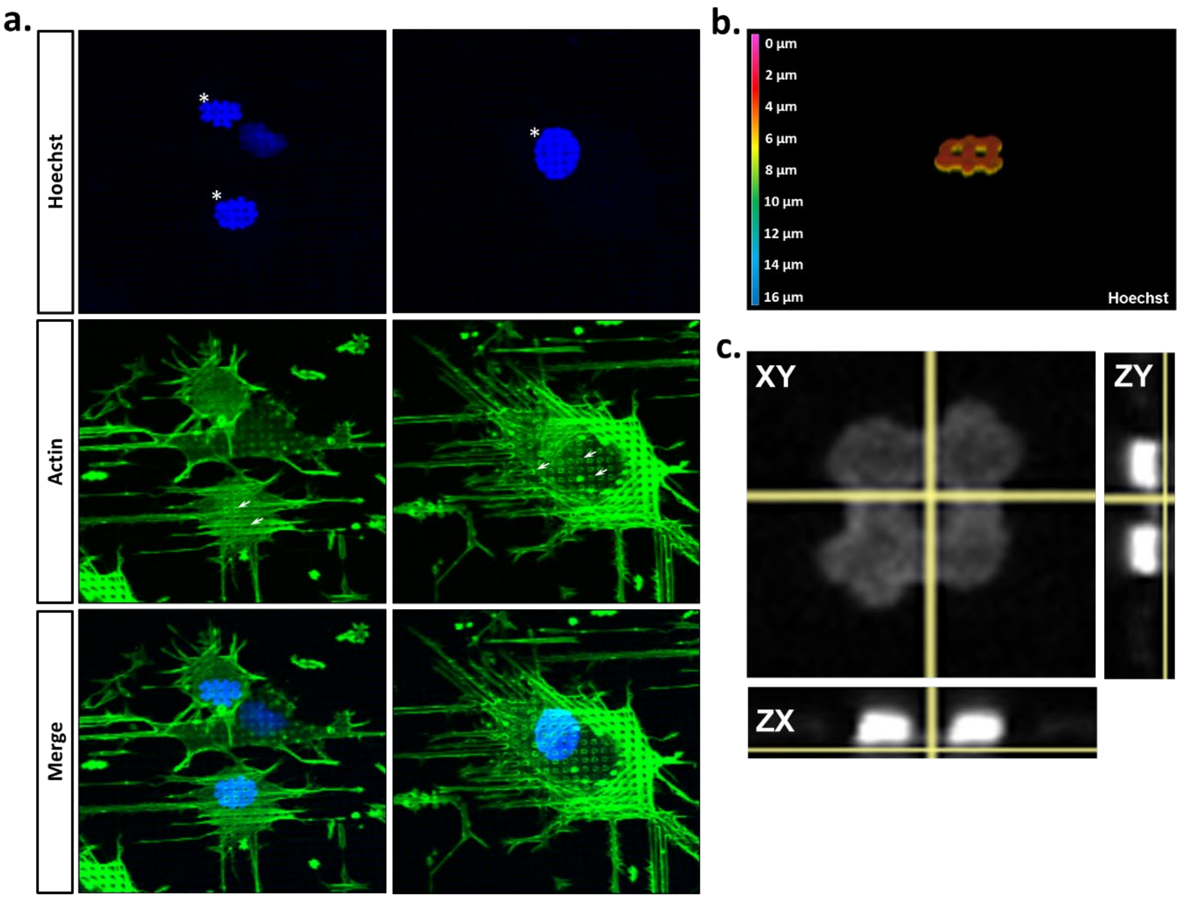

d.

Nuclear shape	Shape descriptor	Defining features
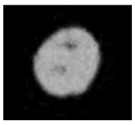	Uniform DNA distribution	• Nuclear circularity > 0.8<br>• No observed chromatin remodelling
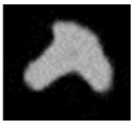	Peripheral nuclear remodelling	• Nuclear circularity < 0.8<br>• No nuclear 'holes'
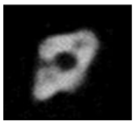	Internal nuclear remodelling	• Nuclear circularity < 0.8<br>• Evident nuclear 'holes'

Figure S5. Micropillars cause DNA displacement within the nuclear compartment as a result of indentation. (a). Representative images of nuclei (blue) and actin (green) of MSCs cultured across micropatterned substrates. Asterisks define severely deformed nuclei containing nuclear indentations whereas arrows highlight regions of actin displacement across the edges of each micropillar. (b). Representative confocal height mapping and image analysis respectively of MSC nuclei, depicting DNA displacement throughout the complete nuclear volume. (d). Defining features used to characterize nuclear indentation patterns resulting from micropatterned culture.

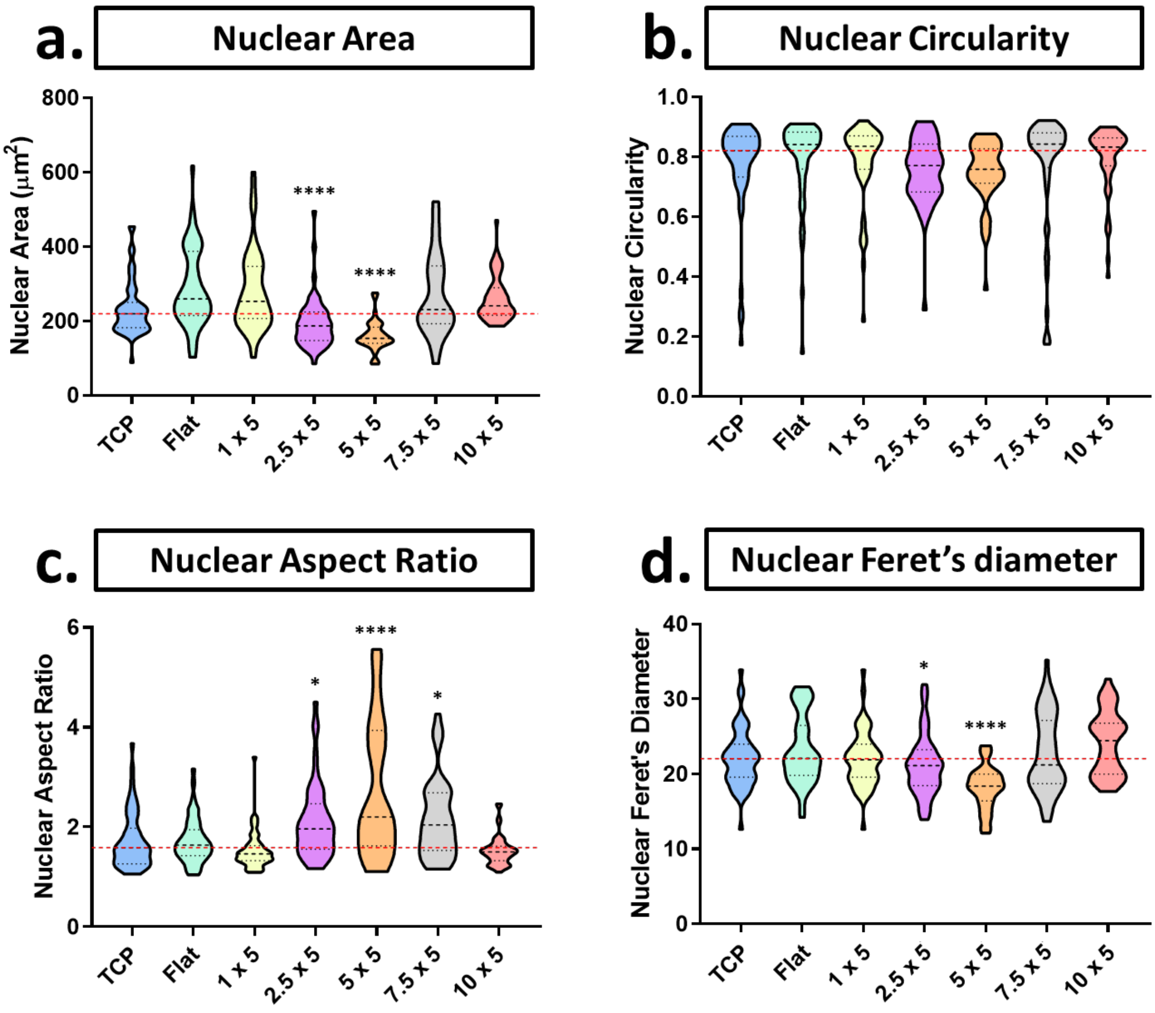

Figure S6. Characterization of MSC nuclear phenotype in response to substrate microtopography. Quantification of nuclear area (a), circularity (b), aspect ratio (c) and feret's diameter (d) for MSCs cultured on 5 μm microstructures of varied width and spacing. All graphs show mean ± SD for three independent MSC donors relative to control samples. Samples were analysed by one-way ANOVA with Tukey post hoc testing. Statistically different samples are denoted by $*p < 0.05$, and $****p < 0.001$.

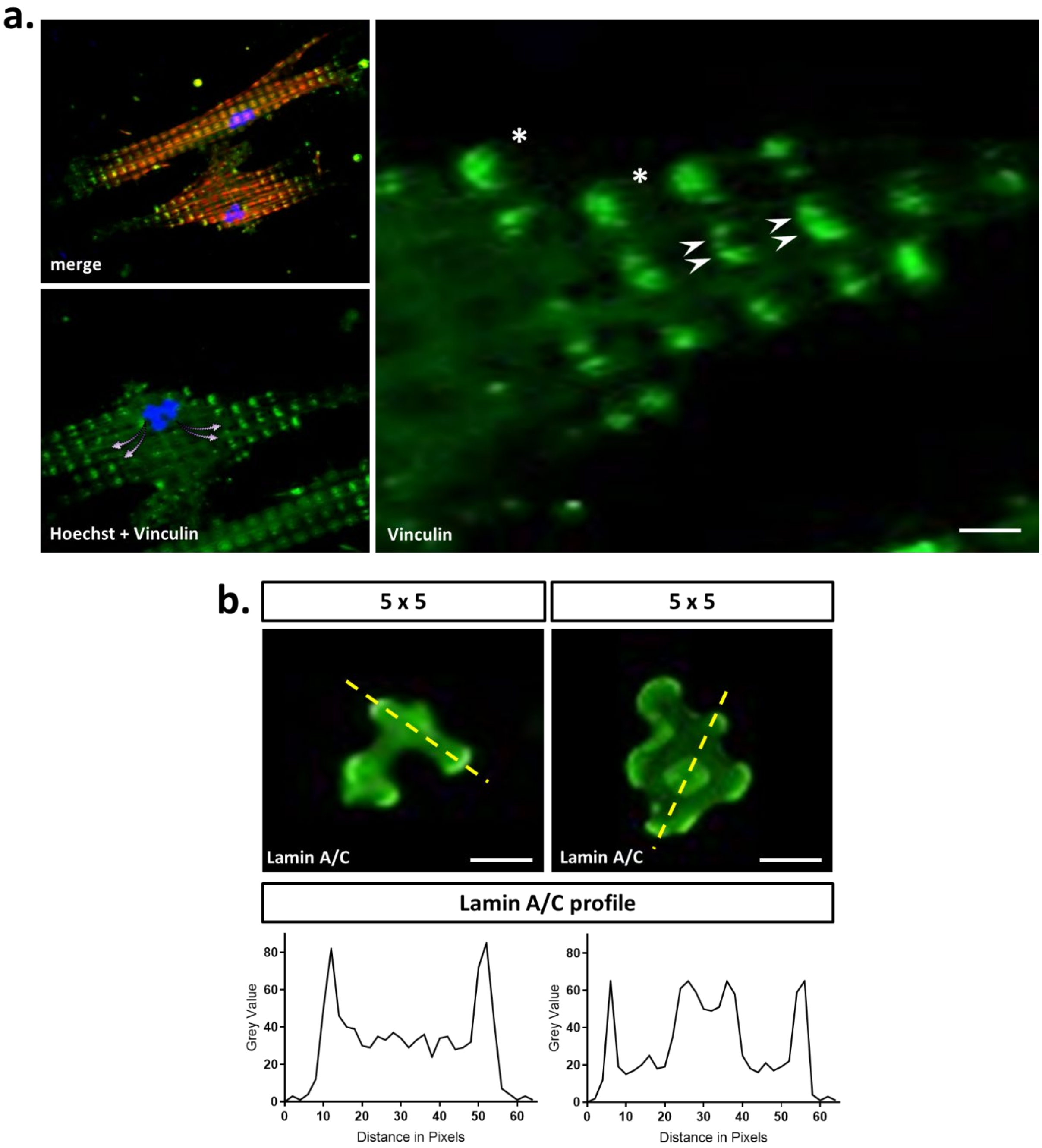

Figure S7. Focal adhesions form around micropillars to enable cell attachment and force dependent nuclear deformations. (a). Immunofluorescence staining of actin (red), nuclei (blue) and vinculin (green) depicting focal adhesion formation across micropillar edges. Asterisks highlight instances of vinculin staining across pillar surface, whereas arrows define non-uniform distribution of vinculin across micropillar edges closest to the nucleus. (b). Graphical representation of lamin A/C distribution across MSCs cultured on the 5 µm micropillars. Scale bars, 5 µm.

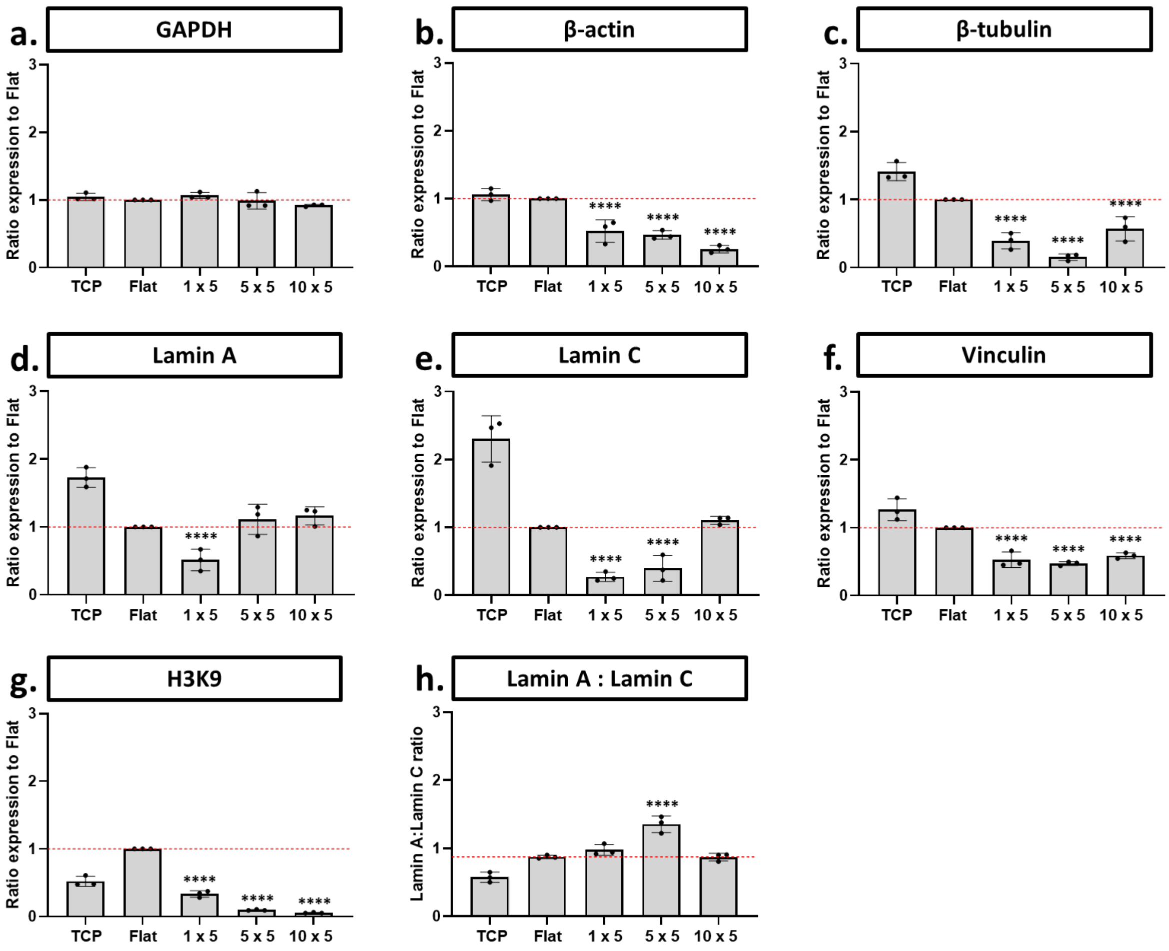

Figure S8. Micropatterned culture stimulates expression changes in cytoskeletal, nuclear and focal adhesion proteins. (a) – (g) densitometry analysis of GAPDH, β-actin, β-tubulin, lamin A, lamin C, vinculin and H3K9 expression profiles between flat (control) and 5 µm micropatterned substrates of varied width and spacing. (h) defines changes in lamin A:C ratio between flat (control) and 5 µm micropatterns of varied width and height. Samples were analysed by one-way ANOVA with Tukey post hoc testing. Statistically different samples are denoted by ****p < 0.001.

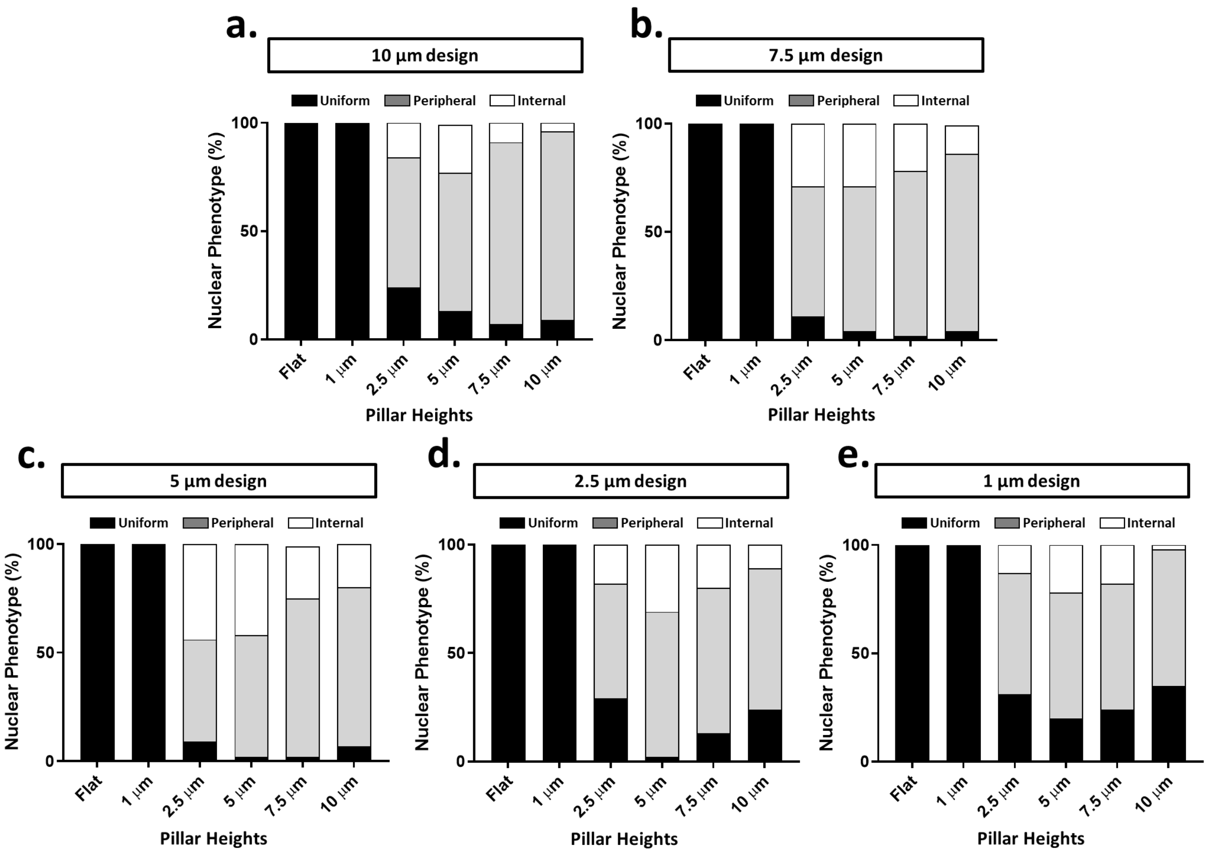

Figure S9. Characterization of MSC nuclear shape in response to changing substrate micro-topography. (a) – (e). Quantification of nuclear shapes observed on each micropillar design tested.

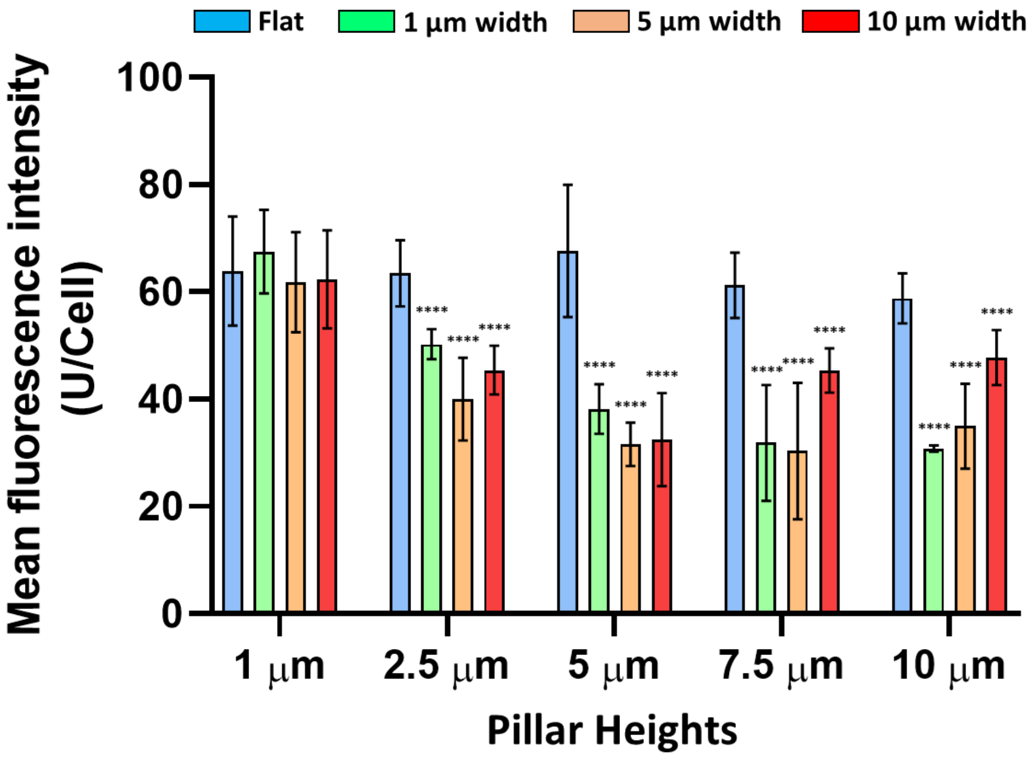

Figure S10. H3K9 expression profile decreases in MSCs on micropillars. Quantification of mean fluorescent intensity in MSCs cultured on varied micropillar heights, widths and spacing. All graphs show mean ± SD for three independent MSC donors relative to control samples. Samples were analysed by one-way ANOVA with Tukey post hoc testing. Statistically different samples are denoted by ****$p < 0.001$.

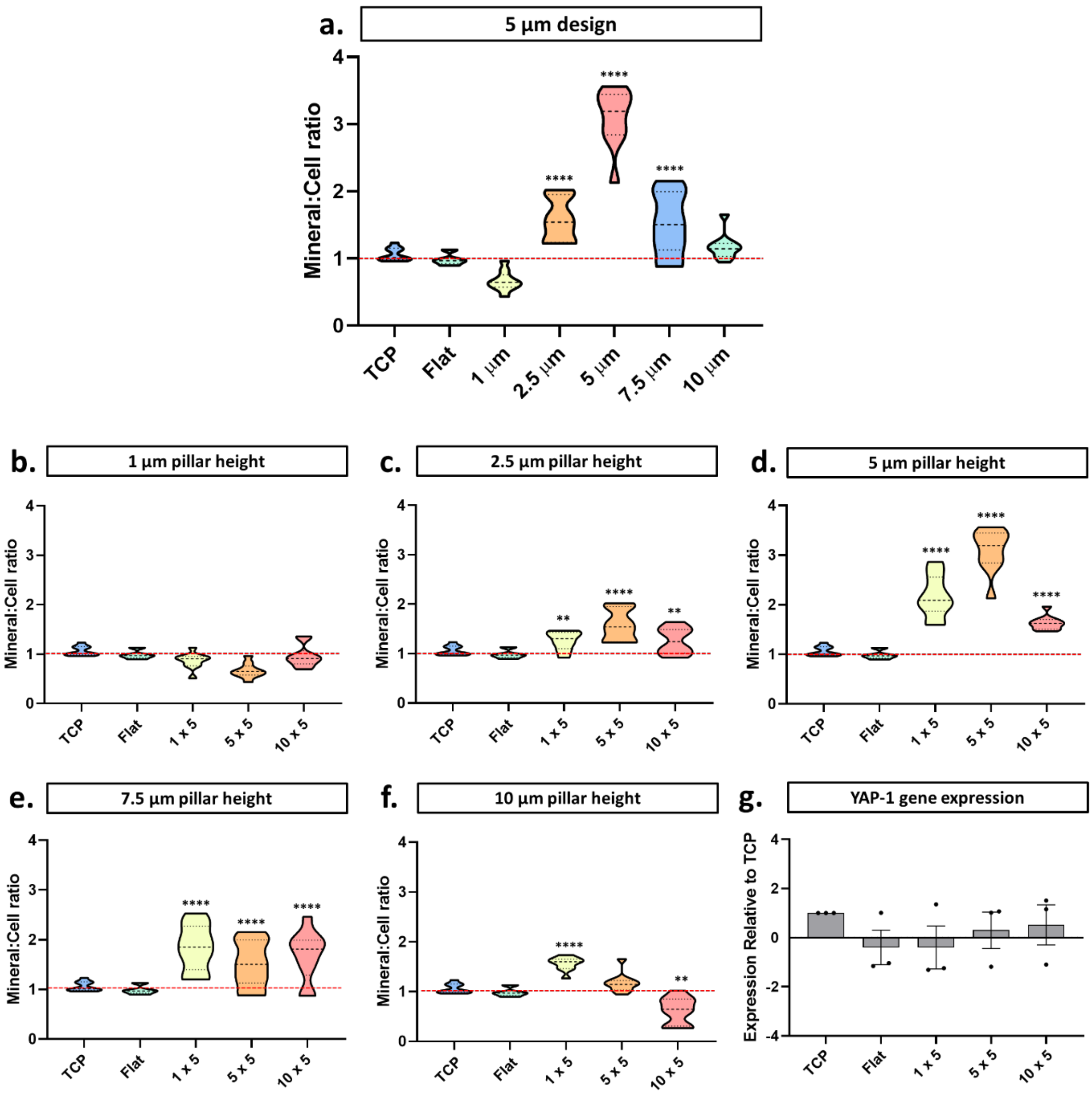

Figure S11. Characterization of enhanced osteogenic potential of micropatterned substrates. (a). Quantification of mineral:cell ratio calculated using the mean fluorescent intensity of Xylenol orange staining. (b) – (f). Quantification of mineral production for MSCs on each micropattern tested. (g) depicts gene regulatory changes for YAP-1 on flat substrates and micropillars as determined via RT-PCR. All graphs show mean ± SD for three independent MSC donors relative to control samples. Samples were analysed by one-way ANOVA with Tukey post hoc testing. Statistically different samples are denoted by $**p < 0.01$ and $****p < 0.001$.

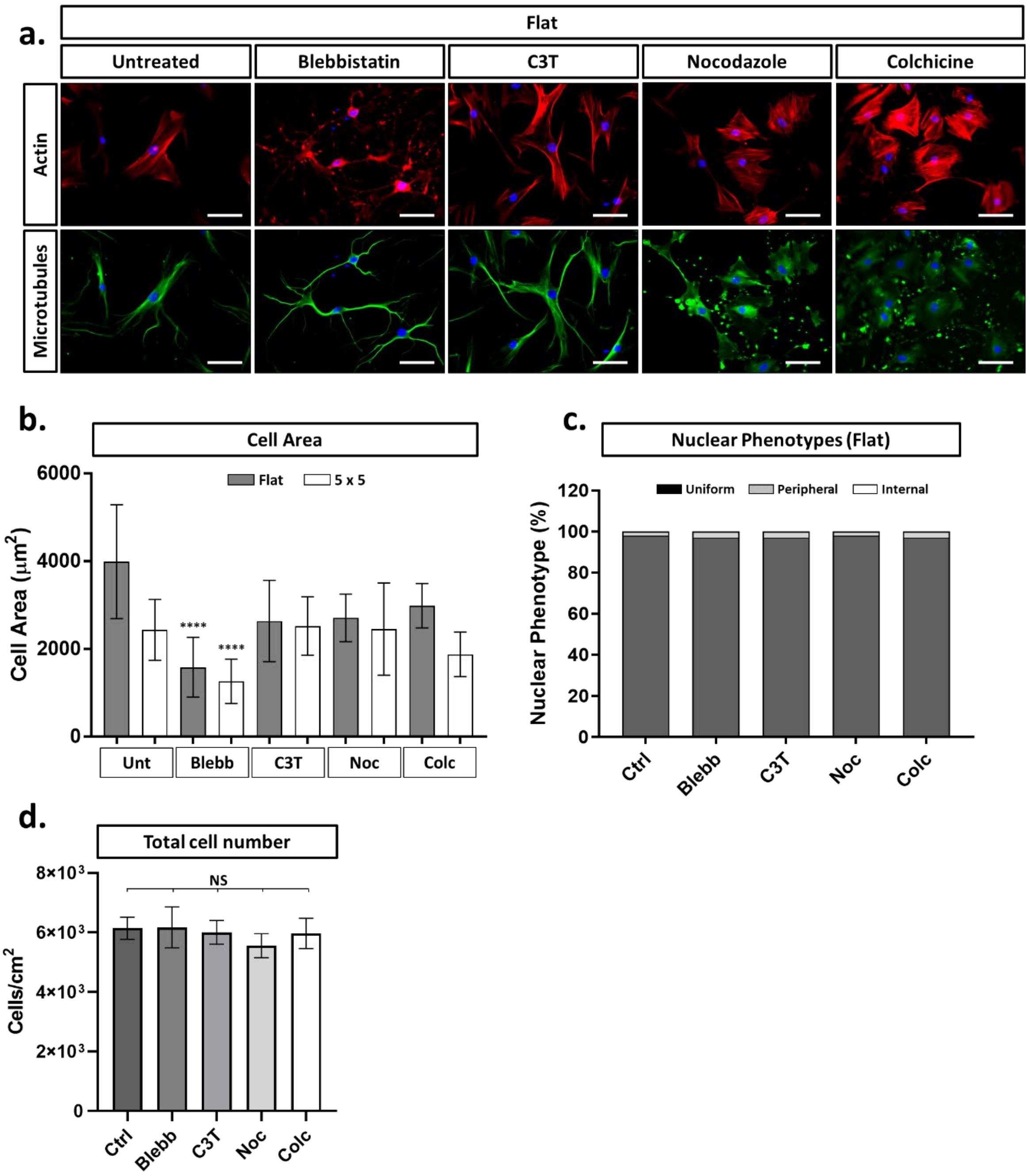

Figure S12. Characterization of phenotypic changes resulting from treatment with actin and microtubule inhibitors. (a). Representative images of actin (red), microtubules (green) and nuclei (blue) in MSCs cultured with microtubule and actin inhibitors on flat substrates. Scale bars, 50 µm. (b) Quantification of cell area following inhibitor addition. (c) Quantification of observed nuclear phenotypes following drug addition on flat substrates. (d) Quantification of total cell numbers across 5x5 grid designs following drug supplemented differentiation. All graphs show mean ± SD for three independent MSC donors relative to control samples. Samples were analysed by one-way ANOVA with Tukey post hoc testing. Statistically different samples are denoted by $^{**}p < 0.01$ and $^{****}p < 0.001$.

Supplementary Table 1

Supplementary Table 1. qPCR forward and reverse primer sequences used in this study.

	Forward 5' – 3'	*Reverse 3' – 5'*
GAPDH	ATGGGGAAGGTGAAGGTCG	TAAAAGCAGCCCTGGTGACC
RPS27a	TGGATGAGAATGGCAAAATTAGTC	CACCCCAGCACCACATTCA
PTK2 (FAK)	GCGCTGGCTGGAAAAAGAGCAA	TCGGTGGGTGCTGGCTGGTAGG
YAP1	CTCGAACCCCAGATGACTTC	CCAGGAATGGCTTCAAGGTA
LMNA	CGGTTCCCACCAAAGTTCA	CTCATCCTCGTCGTCCTCAA
Vinculin	CTCGTCCGGGTTGGAAAAGAG	AGTAAGGGTCTGACTGAAGCAT
DNMT1	AGAACGGTGCTCATGCTTACA	CTCTACGGGCTTCACTTCTTG
DNMT3a	GAAGCCGCTGTTACCTCTTG	TCTGCAAGCTGTCTCCCTTT
DNMT3b	CTCGATCAAACAGGGGAAAA	AAGAGGTGTCGGATGACAGG

GAPDH: glyceraldehyde-3-phosphate dehydrogenase. RPS27a: ribosomal protein S27a. PTK2 (FAK): protein tyrosine kinase 2 (focal adhesion kinase). YAP1: yes associated protein 1. LMNA: gene encoding lamin A and Lamin C proteins. Vinculin: protein of the focal adhesion complex. DNMT1: DNA methyl transferase 1. DNMT3a: DNA methyl transferase 3a. DNMT3b: DNA methyl transferase 3b.
